# Novel allelic variation in the *Phospholipase D alpha*1 gene (*OsPLDα1*) of wild *Oryza* species implies to its low expression in rice bran

**DOI:** 10.1038/s41598-020-62649-w

**Published:** 2020-04-20

**Authors:** Amandeep Kaur, Kumari Neelam, Karminderbir Kaur, Ai Kitazumi, Benildo G. de los Reyes, Kuldeep Singh

**Affiliations:** 1 School of Agricultural Biotechnology, Punjab Agricultural University, Ludhiana, Punjab India; 2 School of Biology and Ecology, University of Maine, Orono, Maine United States of America; 3 Present Address: Department of Plant and Soil Science, Texas Tech University, Lubbock, Texas United States of America; 4 Present Address: ICAR- National Bureau of Plant Genetic Resources, New Delhi, India

**Keywords:** Gene expression profiling, Agricultural genetics

## Abstract

Rice bran, a by-product after milling, is a rich source of phytonutrients like oryzanols, tocopherols, tocotrienols, phytosterols, and dietary fibers. Moreover, exceptional properties of the rice bran oil make it unparalleled to other vegetable oils. However, a lipolytic enzyme Phospholipase D alpha1 (OsPLDα1) causes rancidity and ‘stale flavor’ in the oil, and thus limits the rice bran usage for human consumption. To improve the rice bran quality, sequence based allele mining at *OsPLDα1* locus (3.6 Kb) was performed across 48 accessions representing 11 wild *Oryza* species, 8 accessions of African cultivated rice, and 7 *Oryza sativa* cultivars. From comparative sequence analysis, 216 SNPs and 30 InDels were detected at the *OsPLDα*1 locus. Phylogenetic analysis revealed 20 *OsPLDα*1 cDNA variants which further translated into 12 protein variants. The *O. officinalis* protein variant, when compared to Nipponbare, showed maximum variability comprising 22 amino acid substitutions and absence of two peptides and two β-sheets. Further, expression profiling indicated significant differences in transcript abundance within as well as between the *OsPLDα*1 variants. Also, a new *OsPLDα1* transcript variant having third exon missing in it, *Os01t017*2*400-06*, has been revealed. An *O. officinalis* accession (IRGC101152) had lowest gene expression which suggests the presence of novel allele, named as *OsPLDα1-1a* (GenBank accession no. MF966931). The identified novel allele could be further deployed in the breeding programs to overcome rice bran rancidity in elite cultivars.

## Introduction

Rice (*Oryza sativa* L.) bran, a by-product after milling, is composed of pericarp, aleurone, seed coat, nucellus along with the germ and a small portion of endosperm^1,2^. It constitutes about 10% of the weight of rough rice, and is comprised of 12–23% oil, 14–16% protein, and 8–10% crude fibre. The rice bran oil is an oleic–linoleic-type fatty acid and is rich source of vitamin E, thiamin, niacin, and minerals like aluminium, calcium, chlorine, iron, magnesium, manganese, phosphorus, potassium, sodium, and zinc^3^. Further, the presence of *ω*-3 and *ω*-6 fatty acids, high level of unsaponifiables, and high levels of antioxidants (tocopherols, tocotrienols, and γ-Oryzanol) makes it superior to other vegetable oils as well as brightens the prospects of its utilization for humans as functional ingredient to mitigate the life-threatening disorders^4–6^. In addition, physico–chemical properties make it a good quality edible oil^7^. However, removel of husk from the paddy leads to direct contact of air with rice bran layer, which activates endogenous lipase, and results in development of off-flavor in brown rice. Further, decomposition of triacylglycerols (TAGs) in rice bran, immidiately after the process of milling, raise the levels of free fatty acids (FFAs) which makes the rice bran unsuitable for human consumption or for production of edible oil with acceptable quality^8,9^. In addition, rapid degradation and hydrolytic rancidity of rice bran oil limits its use for human consumption.

Rice bran is mainly comprised of TAGs, which act as the primary reserve lipids and occur in the phospholipid membrane bounded oil bodies. The aleuronic layer at maturation is comprised of living cells in which phospholipids are decomposed into fatty acids and some other chemicals by various phospholipid-degrading enzymes during storage. The phospholipid-degrading enzymes viz. phospholipases, acyl hydrolases, and lipid-oxidizing enzymes have been known as important contributors to membrane degradation^10,11^. The treatment of oil bodies, from rice bran fraction, with Phospholipase D (PLD) causes oil bodies disintegration followed by reduction of phosphatidylcholine levels and TAGs decomposition into FFAs^12,13^. Further, the FFAs interact with endosperm starches to reduces the edibility of the rice. In addition, lipoxygenases act on the FFAs which contain a 1, 4-pentadiene structure, such as linoleic and linolenic acids, and lead to their conversion into low molecular-weight volatile products which cause a stale flavor in the product^14,15^. Hence, it has been revealed by the the earlier studies that PLD acts as a trigger for the initiation of lipid decomposition which further leads to deterioration of the rice grain and rice bran fractions.

A total of 17 PLD genes including eight isoforms of *PLDα*, two of *PLDβ*, three of *PLDγ*, two of P*LDξ*, and one isoform each of *PLDκ* and *PLDφ* has been indicated in rice genome database^16^. In these isoforms, protein domain analysis has revealed several conserved domains, including the HKD (HxKxxxxD) domains (also known as PLD-C1 and PLD-C2 domains), having hydrolytic activity; the calcium/lipid-binding domain (C2 domain), resonsible for regulation of Ca^2+^-dependent enzyme activity through binding to Ca^2+^; and (3) the PX (phox consensus sequence) and PH (pleckstrin homology) domains, located at the N-terminus of Ca^2+^ independent PLDs in place of the C2 domain of Ca^2+^ dependent PLDs^17^. From the rice bran fraction, a PLD protein (designated RPLD1, synonymous with OsPLDα1) has been purified and is found to be responsible for rice bran oil rancidity^18^. Suzuki *et al*. (2011) cloned the sequence of *OsPLDα1* from *O. sativa japonica* cv. Nipponbare. This gene is 6.28-kb in size including promoter region and is located on the chromosome 1 of rice^19^. The expression profiling reveals that most PLD-encoding genes are differentially expressed in many plant tissues, and during various developmental stages, suggesting their involvement in multiple developmental processes^20^. However, studies using transgenics have clarified that the suppressed Os*PLD*α*1* expression results in the improvement of grain and bran stability. In addition, this gene has been reported to be unnecessary for seed maturation or germination^21^.

Although various stabilization methods are available to inhibit the Os*PLD*α*1* lipolytic process^22,23^, such methods only lead to partial inactivation; reduce the nutritional value of rice bran; and increase the time stringency for treatment and cost of oil production^24^. Thus, a profitable substitute is required to reduce the rice bran rancidity. The use of breeding techniques could increase the rice bran stability against lipolytic process if genetic differences exist for this trait. However, the hassle of diminished gene pool in cultivated germplasm is specifically relevant in self pollinated crops where the degree of genetic variation in cultivars can be less than 5% of the total variation in natural populations. As a result of the selection deployed by humans during domestication in favour of desired traits, the acquired early varieties carry only a small portion of the genetic diversity available in wild species^25^. Hence, for the current study, we chose a representative subset of the wild rice germplasm as it constitutes a major gene pool for rice improvement^26,27^. Further, the allele mining technique have been successfully employed in wild species to find important variations at various *loci* including *Badh*2, *OsC1, Pi ta*, NBS-LRR class R-genes, *Adh2, wx* locus, and *Rc* locus^28–34^. However, thus far, wild germplasm of rice has not been assessed for the variability at *OsPLDα1* locus.

Therefore, in the current study, a detailed analysis of DNA sequence variation at the *OsPLDα1* locus (*Os01g0172400*) was performed in a panel of wild and cultivated rice (*Oryza* spp.) to identify the novel sources of alleles with lower or null activity of the enzyme. Further, validation of the identified *OsPLDα1* allelic variants was conducted using quantitative reverse-transcription expression analysis.

## Results

### SNPs within the coding region of *OsPLDα1*

The complete coding region of *OsPLDα1*, in all the wild *Oryza* accessions and cultivars (Table 1), was found to be ~2248 bp long and was comprised of three exons. A total of 105 SNPs and 2 insertions were identified in the coding region of *OsPLDα1* gene across wild species accessions and cultivars (see Supplementary Table S1), using multiple sequence alignments. Within the first exon of *OsPLDα1* gene (located on the gene from nucleotide position 353 to 460), only one nucleotide change (T373C) was observed across the accessions of *O. officinalis, O. australiensis, O. punctata*, *O. minuta*, and *O. latifolia* spp.

**Table 1:** Selected *Oryza* spp. accessions for allele mining at *OsPLDα1* locus.

S. No.	Species	Accession #	Genome	Country of origin
1	*O. glaberrima*	IRGC100854	AA	Congo
2	*O. glaberrima*	IRGC101800	AA	Senegal
3	*O. glaberrima*	IRGC102196	AA	Liberia
4	*O. glaberrima*	IRGC102489	AA	Liberia
5	*O. glaberrima*	IRGC102512	AA	Liberia
6	*O. glaberrima*	IRGC102600b	AA	Liberia
7	*O. glaberrima*	IRGC102925	AA	Burkina Faso
8	*O. glaberrima*	IRGC103750	AA	Nigeria
9	*O. barthii*	IRGC100117	AA	Mali
10	*O. barthii*	IRGC101317	AA	Guinea
11	*O. barthii*	IRGC104102	AA	Chad
12	*O. barthii*	IRGC 105990	AA	Cameroon
13	*O. barthii*	IRGC106239	AA	Nigeria
14	*O. barthii*	IRGC106294	AA	Chad
15	*O. nivara*	CR100008	AA	India
16	*O. nivara*	CR100400	AA	India
17	*O. nivara*	CR100126	AA	India
18	*O. nivara*	CR100429	AA	India
19	*O. nivara*	IRGC80547	AA	India
20	*O. nivara*	IRGC81847	AA	India
21	*O. nivara*	IRGC92713	AA	Cambodia
22	*O. nivara*	IRGC92930	AA	Combodia
23	*O. nivara*	IRGC100189	AA	Malaysia
24	*O. nivara*	IRGC106397	AA	India
25	*O. rufipogon*	CR100013	AA	India
26	*O. rufipogon*	IRGC80610	AA	India
27	*O. rufipogon*	IRGC81976	AA	Indonesia
28	*O. rufipogon*	IRGC83823	AA	Vietnam
29	*O. rufipogon*	IRGC89224	AA	Combodia
30	*O. rufipogon*	IRGC99551	AA	Vietnam
31	*O. rufipogon*	IRGC103308	AA	Taiwan
32	*O. rufipogon*	IRGC104308	AA	Myanmar
33	*O. rufipogon*	IRGC104867	AA	Thailand
34	*O. rufipogon*	IRGC105491	AA	Malaysia
35	*O. rufipogon*	IRGC105569	AA	Cambodia
36	*O. rufipogon*	IRGC105902	AA	Bangladesh
37	*O. rufipogon*	IRGC106162	AA	Laos
38	*O. rufipogon*	IRGC106336	AA	Cambodia
39	*O. rufipogon*	IRGC106433	AA	Vietnam
40	*O. rufipogon*	IRGC113652	AA	Vietnam
41	*O. longistaminata*	IRGC101200	AA	Nigeria
42	*O. longistaminata*	IRGC104301	AA	Gambia
43	*O. longistaminata*	IRGC105206	AA	Ethiopia
44	*O. meridionalis*	IRGC101146	AA	Australia
45	*O. glumaepatula*	IRGC100184	AA	Cuba
46	*O. glumaepatula*	IRGC104387	AA	Brazil
47	*O. officinalis*	IRGC101152	CC	Brunei
48	*O. officinalis*	IRGC105674	CC	Indonesia
49	*O. officinalis*	IRGC106501	CC	Indonesia
50	*O. australiensis*	IRGC105275	EE	Australia
51	*O. punctata*	IRGC101434	BBCC	Tanzania
52	*O. punctata*	IRGC105158	BBCC	Kenya
53	*O. minuta*	IRGC101100	BBCC	Philippines
54	*O. minuta*	IRGC101128	BBCC	Philippines
55	*O. latifolia*	IRGC100165	CCDD	Guatemala
56	*O. latifolia*	IRGC105139	CCDD	Guatemala

Codes: IRGC represents the wild species accessions from the International Rice Genetic Consortium, IRRI, Philippines; CR represents accessions from National Rice Research Institute, Cuttack, India.

**Figure 1:**
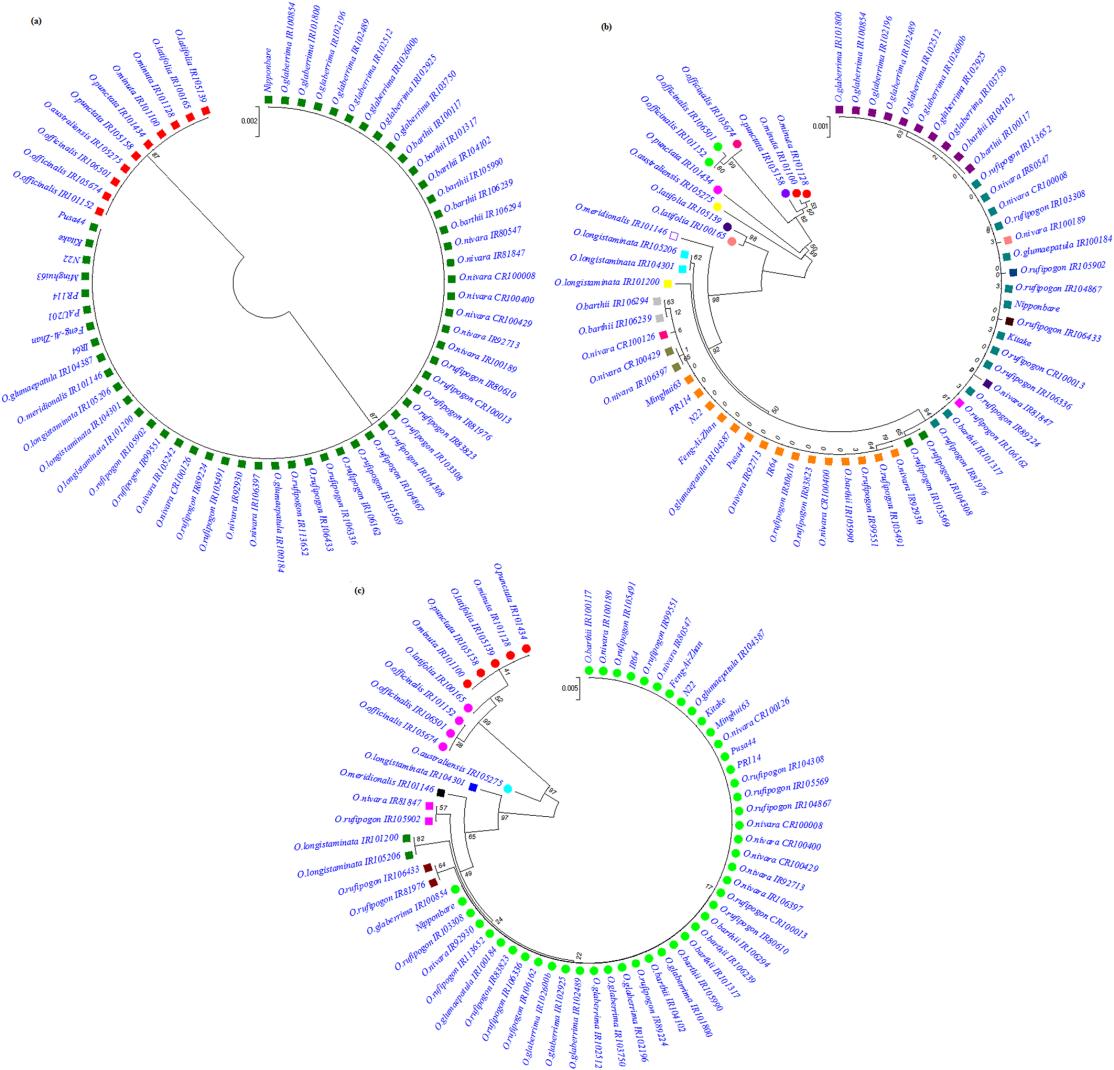
Evolutionary relationship across different wild species accessions and cultivars based on the nucleotide sequence of *OsPLDα1* exons (**a**) first exon, **(b)** second exon, **(c)** third exon using a neighbor –joining algorithm calculated by boot-strap value of 1000 replicate.

In addition, these species also had an insertion of nucleotide A at position 459. On the contrary, all the accessions belonging to ‘AA’ genome species and selected cultivars showed no polymorhism at the first exon and fall in the same cluster along with reference sequence of Nipponbare (Fig. 1a). The second exon of *OsPLDα1*, located on the gene from nucleotide position 1001 to 2897, was found to harbor the maximum variability (87 SNPs and an insertion of T_1927_) in the coding region. The detected SNPs were comprised of 65 transition changes and 22 transversions. G1141A was observed as the most frequent SNP followed by G1607A. Across all the selected wild species accessions and cultivars, maximum number of SNPs (73) were present in the species belonging to the *O. officinalis* complex (*O. officinalis, O. australiensis, O. punctata*, *O. minuta*, and *O. latifolia*). Moreover, *O. officinalis* spp. having 52 SNPs and an insertion of A at position 1927 was found most polymorphic among all (Fig. 1b). Of the total SNPs identified in *O. officinalis* complex, a few SNPs were also observed in the two AA genome species viz. *O. meridionalis* (T1135C, T1153C, T1207C, C1156T, A1747, A2099T, A2855G, and C1810T) and *O. longistaminata* (A1639G, A1747G, A2099T, and A2855G). AA genome species were found to carry only 27% of the total variations detected at second exon. Cultivars including Pusa44, Feng-Ai-Zhan, Minghui63, PR114, IR64, and N22 were observed to have two nucleotide changes, G1141A and G1607A, in the second exon, however, cultivar Kitake showed no polymorhism and, thus had more relatedness to the Nipponbare when compared to rest of the cultivars.

A total of 17 polymorphic sites were reported within the third exon (located on the gene from nucleotide position 3376 to 3618) of the gene. Accessions of *O. punctata, O. latifolia, O. minuta and O. officinalis* spp. were observed to harbor most of the variability present on third exon, and consequently were found least related to the Nipponbare sequence (Fig. 1c). However, all the cultivars were found monomorphic for the third exon of *OsPLDα1*.

### SNP analysis of UTRs of *OsPLDα1* gene

Untranslated regions (UTRs) play an importance role in stabilizing RNA and regulating the transcript expression. Moreover, variations within the 5′ UTR are also known to alter the transcription rate. Similar to the Nipponbare, UTR in all the wild species accessions and cultivars was found separated by an intronic region, however, variations in length and nucleotide sequences of two separeted UTRs (UTR1 and UTR2) were observed in different accessions due to the presence of SNPs and InDels. In comparison to the 142 nucleotide long UTR1 in Nipponbare, length of the UTR1 measured 139 nucleotides in *O. officinalis* and *O. minuta* spp. (see Supplementary Fig. S1) due to the presence of 5 InDels of 1 (Insertion of T), 3 (Deletion of TCT), 5 (Insertion of GCCTC), 1 (Deletion of T), and 5 nucleotides (Deletion of CCTCC) at positions 4, 58, 80, 102, and 124, respectively. Additionaly, SNPs A40G, C50A, C101G, C102T, and C141T were also observed in the UTR1 region of these species. Across all the sequenced rice cultivars and AA genomic wild species, an InDel of 3 nucleotides (Inserion of CTC) at position 99 was observed in the UTR1. Moreover, cultivars Feng-Ai-Zhan, Pusa44, IR64, and Minghui 63 also had a deletion of C at nucleotide position 69. In UTR2, a deletion of 10 nucleotides (AATCCAAATC) at nucleotide position 16, was detected in *O. officinalis, O. minuta, O. punctata*, and *O. australiensis* spp., when compared to the Nipponbare (see Supplementary Fig. S2). In addition, 3 SNPs, G5A, A21T, A22C and T23A were observed in these species as well. However, all the accessions of AA genomic species and all the cultivars were found monomorphic for UTR2 nucleotide sequence.

### SNPs within intronic regions

**Figure 2:**
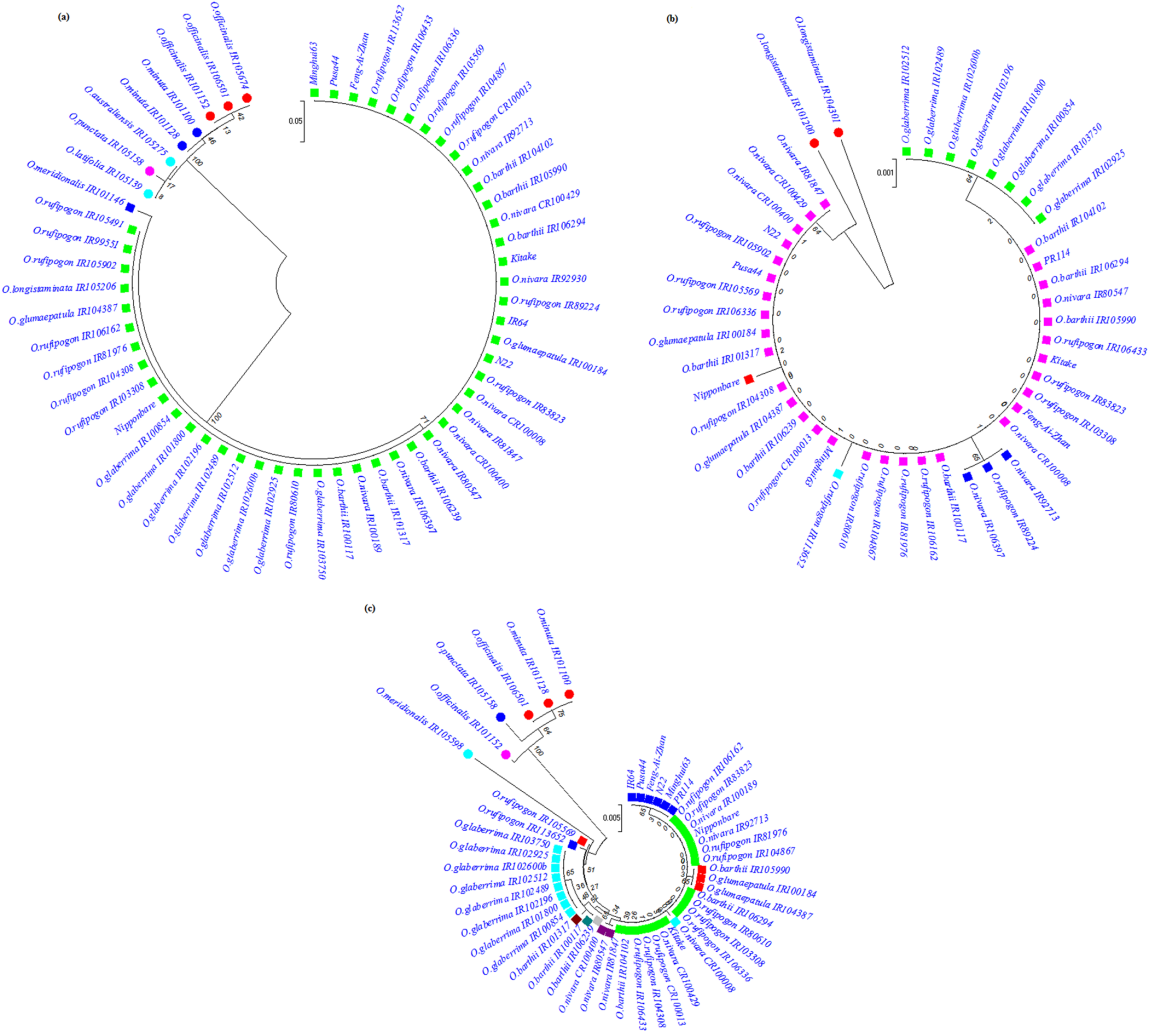
Evolutionary relationship across different wild species accessions and cultivars based on the nucleotide sequence of *OsPLDα1* introns (**a**) first intron, **(b)** second intron, **(c)** third intron, using a neighbor –joining algorithm calculated by boot-strap value of 1000 replicate.

Across the wild species and cultivars, we detected 101 SNPs and 22 InDels in the intronic region of *OsPLDα1* gene, in comparison to the Nipponbare (see Supplementary Table S2). Within the first intron (located on the gene from nucleotide position 143 to 315), 50 nucleotide changes were identified and most of them (45) were detected in the accessions of *O. officinalis* (IR101152, IR105674, IR106501)*, O. minuta* (IR101100, IR101128)*, O. punctata* (IR105158), *O. australiensis* (IR105275), and *O. latifolia* (IR105139) spp. The remaining 5 SNPs (C187T, C257G, A286T, T289C, and G309T) were detected in the *O. meridionalis* accession (IR101146). In addition to the nucleotide changes, 4 InDels of 9 (+TCGCTGTAC_222–230_), 11 (+ATTTCTTATCC_147–157_), 13 (+ATCCTCGCTTACC_147–159_), and 6 (−AGGTAG_176–181_) nucleotides, were also observed in the species belonging to the *O. officinalis* complex. Across the cultivars, only a single InDel of 1 nucleotide long (+G_315_) was detected in Pusa44, Minghui63, and Feng-Ai-Zhan. No SNP or InDel was detected across the accessions of *O. glaberrima, O. barthii, O. nivara, O. glumaepatula*, and *O. longistaminata*. Phylogenetic tree generated from the nucleotide sequence of first intron showed that the accessions of selected species and cultivars fall into two clades (Fig. 2a).

Second intron (located on the gene from nucleotide position 461 to 1000) sequence was only available for AA genome species and cultivars, and could not be obtained for the rest of species even after repeated efforts. In total, 11 SNPs and an Indel of 4 nucleotides were detected on this intron. A467G SNP was found as the most frequent as it was detected across all the AA genome species and rice cultivars. *O. longistaminata* accessions (IR104301 and IR101200) harbour maximum variability on the second exon, and thus were found most distant to the Nipponbare (Fig. 2b). The third intron (cover 2898 to 3375 nucleotide position on the gene) carried 40 nucleotide changes and 6 InDels. Among AA genome species, *O. meridionalis* had maximum number of SNPs; however *O. officinalis* was observed to have maximum variability (SNPs and InDels) across all the wild species. Within the third intron, two large InDels of 18bp (+ATGCATCAGAGATCATTT) and 30bp (CTAATGATCAAGCTAGTAACTTCATCTCCT) were detected from the nucleotide positions 2988 to 3006 and from 3295 to 3324, respectively. Accessions of *O. officinalis, O. minuta*, and *O. punctata* were falling in the same cluster, and were found least related to the Nipponbare (Fig. 2c).

### *OsPLDα1* cDNA and protein variants

**Figure 3:**
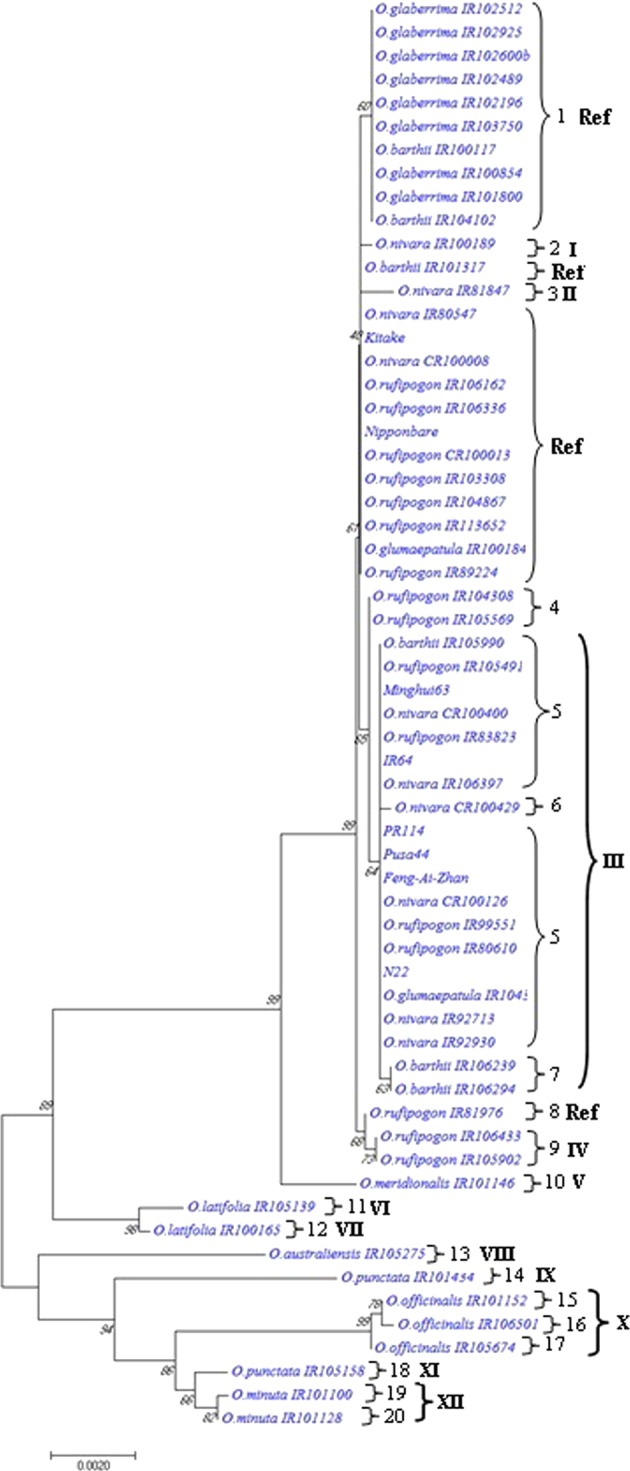
Phylogenetic relationship of *OsPLDα1* across Nipponbare, wild species accessions, and cultivars of rice based on the nucleotide sequence data of cDNA. Phylogenetic tree was generated using a neighbor - joining algorithm calculated by boot-strap value of 1000 replicate. The number 1–20 indicates 20 *OsPLDα1* variants based on the nucleotide sequences of cDNA while the numbers I to XII indicate protein variants. ‘Ref’ denotes the nucleotide sequences variants which translates into the amino acid sequence same as that of the reference OsPLDα1 protein sequence of Nipponbare.

A panel of 63 *OsPLDα1* cDNA sequence assemblies from wild *Oryza* species accessions and cultivars, each containing ~2248 bp were analyzed. Phylogenetic analysis revealed the presence of *OsPLDα1* variants in 48 accessions from 11 wild *Oryza* spp., 8 accessions of *O. glaberrima*, and 7 *Oryza sativa* cultivars (Fig. 3). These *OsPLDα1* variants were further classified into two major clusters that distinguish AA genomic spp. (*O. glaberrima, O. barthii, O. nivara, O. rufipogon, O. longistaminata, O. meridionalis*, and *O. glumaepatula*) from other genomic spp. (*O. officinalis*, *O. australiensis, O. punctata*, and *O. minuta*). Accessions of *O. latifolia* were falling in between the two clusters. The reference sequence of Nipponbare showed more closeness to *japonica* cultivar Kitake than to *indica* cvs. Minghui 63, IR 64, PR 114, Pusa 44, Feng-Ai-Zhan, and N22. The analysis revealed that polymorhic sites were frequently observed in the wild spp. having genome other than AA genome. A total of 20 *OsPLDα1* variants were identified based on the nucleotide variations in the cDNA sequences of selected wild species accessions and cultivars.

To determine if the detected nucleotide variations in the coding region of the gene further lead to any alterations in the gene, the cDNA structures of the representative *Oryza* accessions were aligned with the Nipponbare (see Supplementary Fig. S3). The results revealed that all AA genomic spp. were having the similar number of exons as of the Nipponbare. Exon1 and Exon 3 were found to be of same length in all the studied species whereas Exon2 showed alterations in the species belonging to *O. officinalis* complex. At the end of first exon, a gap of 193bp (from nucleotide position 109 to 301) was detected in *O. australiensis, O. punctata, and O. latifolia* accessions while a smaller gap of 77 bp (from nucleotide position 109 to 184) was detected in the *O. minuta* and *O. officinalis* accessions. Interestingly, *O. officinalis* accession had an additional gap within the exon2 from the nucleotide position 1081 to 1137.

Further, from comparative sequence analysis, 107 nucleotide changes (105 SNPs and 2 insertions) were observed across the exons (see Supplementary Table S1). The identified SNPs included 81 transitions and 24 transversions, while G/A transition was the most common (23.80%). Of the identified nucleotide changes, 16 SNPs and 2 insertions were found to be non-synonymous SNPs/indels that really have the potential to become a novel functional alleles (Table 2). The identified 20 *OsPLDα1* cDNA variants translated into 12 OsPLDα1 proteins variants (designated as I to XII) and the amino acids substitutions in these variants, in comparison to the reference protein sequence of Nipponbare, have been shown in the Table 3. The proteins predicted from *O. officinalis* complex clade had more polymorphic amino acids in comparison to the clade containing AA genome species. In addition to the amino acid substitutions across different regions of the the protein variants, 15 amino acids long peptide (KFVEGIEDTVGKGAT) was found missing at 36^th^ position of the variants VI-XII (see Supplementary Fig. S4). Another 18 amino acids long peptide (RIVSFVGGLDLCDGRYDT) at position 336 was found missing only in the variant X (see Supplementary Fig. S5). As a result, X protein variant which comprised of three accessions of *O. officinalis* (IR101152, IR106501, and IR105674) was having maximum number, twenty two, of amino acid substitutions, and had both the peptides missing.

**Table 2:** Translational modification sites observed at *OsPLDα1* locus across the wild *Oryza* species accessions and *Oryza* cultivars as compared to the Nipponbare reference sequence.

S.No.	Position^#^	Alleles*	Codon change	S.No.	Position^#^	Alleles*	Codon change
1	373	T/C^†^	—	54	1964	T/C^†^	—
2	1027	G/A^†^	—	55	1967	C/T^†^	—
3	1043	G/A^†^	—	56	1997	G/A^†^	—
4	1060	T/C^†^	—	57	2048	T/C^†^	—
5	1084	T/C^†^	—	58	2077	C/A^††^	Thr395Asn
6	1087	A/T^††^	—	59	2081	A/G^†^	—
7	1087	A/C^††^	—	60	2084	A/G^†^	—
8	1090	G/C^††^	—	61	2087	C/A^††^	—
9	1118	A/G^†^	Ile76Val	62	2087	C/T^†^	—
10	1120	C/T^†^	—	63	2089	A/C^††^	Lys399Thr
11	1121	A/G^†^	Asn77Asp	64	2099	A/T^††^	—
12	1123	C/T^†^	—	65	2102	T/A^††^	—
13	1135	T/C^†^	—	66	2108	G/A^†^	—
14	1141	G/A^†^	—	67	2174	G/A^†^	—
15	1153	T/C^†^	—	68	2210	T/A^††^	—
16	1156	C/T^†^	—	69	2232	G/A^†^	—
17	1162	T/C^†^	—	70	2258	T/C^†^	—
18	1168	T/A^††^	—	71	2297	A/C^††^	—
19	1207	T/C^†^	—	72	2335	C/T^†^	Pro481Leu
20	1210	G/A^†^	—	73	2363	T/C^†^	—
21	1243	C/T^†^	—	74	2414	A/T^††^	—
22	1258	T/C^†^	—	75	2456	A/T^††^	—
23	1273	C/T^†^	—	76	2498	C/T^†^	—
24	1275	G/C^††^	Arg128Thr	77	2558	G/T^††^	—
25	1282	C/T^†^	—	78	2582	C/T^†^	—
26	1303	C/T^†^	—	79	2627	G/A^†^	—
27	1308	C/T^†^	Pro139Leu	80	2636	A/C^††^	—
28	1348	C/T^†^	—	81	2657	A/T^††^	—
29	1369	T/A^††^	Asn159Lys	82	2675	G/A^†^	—
30	1386	G/A^†^	—	83	2715	C/T^†^	—
31	1387	C/T^†^	Arg165His	84	2727	G/A^†^	—
32	1450	C/G^††^	—	85	2738	G/A^†^	—
33	1516	C/T^†^	—	86	2794	C/G^††^	Ala634Gly
34	1570	A/G^†^	—	87	2795	T/C^†^	—
35	1607	G/A^†^	Glu239Lys	88	2855	A/G^†^	—
36	1619	G/A^†^	Val729Ile	89	3407	A/G^†^	—
37	1639	A/G^†^	—	90	3425	G/A^†^	—
38	1723	T/C^†^	—	91	3446	T/C^†^	—
39	1747	A/G^†^	—	92	3452	C/T^†^	—
40	1753	G/A^†^	—	93	3461	C/T^†^	—
41	1760	T/C^†^	—	94	3481	C/T^†^	—
42	1762	G/A^†^	—	95	3485	G/A^†^	—
43	1788	A/C^††^	Glu299Ala	96	3488	C/T^†^	—
44	1789	A/T^††^	—	97	3494	A/G^†^	—
45	1808	G/A^†^	Asp306Asn	98	3497	A/G^†^	—
46	1810	C/T^†^	—	99	3503	T/G^††^	—
47	1825	A/G^†^	—	100	3549	G/A^†^	—
48	1850	G/A^†^	Gly320Ser	101	3551	A/C^††^	—
49	1858	T/C^†^	—	102	3553	T/C^†^	Met728Thr
50	1870	G/A^†^	—	103	3554	G/A^†^	—
51	1873	G/A^†^	—	104	3560	T/C^†^	—
52	1879	A/G^†^	—	105	3563	T/C^†^	—
53	1962	A/G^†^	—				

*In addition to the identified SNPs, 2 insertions including A at nucleotide position 459 and T at nucleotide position 1927 were also detected. Insertion of A_459_ led to deletion of KFVEGIEDTVGVGKGAT peptide at amino acid position 36. Insertion of T_1927_ led to amino acid substitutions viz. Leu345Phe, Pro346Ala, Agn347Lys, Gln348Pro, Ser350Leu, Gln351Pro, Gln352Thr, Arg353Lys, Gln372Glu, Tyr373Asp, His374Ser, Ser375Gln, Phe377Arg, and Arg378Trp and a deletion of RIVSFVGGLDLCDGR peptide at amino acid position 354.

^#^The SNP position was calculated relative to the reference sequence of *OsPLDα1* in Nipponbare (RAP locus ID *Os01g0172400*).

Transitions^†^ and transversions ^††^observed as nucleotide substitutions.

**Table 3:** Amino acid variations among 20 *OsPLDα1* variants.

Protein^a^	Ref.^c^	I	II	III	IV	V	VI	VII	VIII	IX	X	XI	XII
Variant^b^	1, 4, 8	2	3	5, 6, 7	9	10	11	12	13	14	15, 16, 17	18	19, 20
76	I	I	I	I	I	I	I	I	I	I	V	V	V
77	N	N	N	N	N	N	N	N	N	N	D	N	N
128	R	R	R	R	R	R	R	R	T	T	T	T	T
139	P	P	P	P	P	P	P	P	P	P	P	L	P
159	N	N	N	N	N	N	N	N	K	N	N	N	N
165	R	R	H	R	R	R	R	R	R	R	R	R	R
239	E	E	E	K	E	E	E	E	E	E	E	E	E
243	V	V	V	V	V	V	V	V	I	V	V	V	V
299	E	E	E	E	E	E	E	E	A	A	A	A	A
306	D	D	D	D	N	D	D	D	D	D	D	D	D
320	G	G	G	G	G	G	G	G	S	G	G	G	G
345	L	L	L	L	L	L	L	L	L	L	F	L	L
346	P	P	P	P	P	P	P	P	P	P	A	P	P
347	N	N	N	N	N	N	N	N	N	N	K	N	N
348	Q	Q	Q	Q	Q	Q	Q	Q	Q	Q	P	Q	Q
350	S	S	S	S	S	S	S	S	S	S	L	S	S
351	Q	Q	Q	Q	Q	Q	Q	Q	Q	Q	P	Q	Q
352	Q	Q	Q	Q	Q	Q	Q	Q	Q	Q	T	Q	Q
353	R	R	R	R	R	R	R	R	R	R	K	R	R
372	Q	Q	Q	Q	Q	Q	Q	Q	Q	Q	E	Q	Q
373	Y	Y	Y	Y	Y	Y	Y	Y	Y	Y	D	Y	Y
374	H	H	H	H	H	H	H	H	H	H	S	H	H
375	S	S	S	S	S	S	S	S	S	S	Q	S	S
377	F	F	F	F	F	F	F	F	F	F	R	F	F
378	R	R	R	R	R	R	R	R	R	R	W	R	R
395	T	T	T	T	T	N	N	N	N	N	N	N	N
399	K	N	K	K	K	K	K	K	T	K	T	T	T
481	P	P	P	P	P	P	P	P	L	P	P	P	P
634	A	A	A	A	A	A	G	A	G	G	G	A	A
728	M	M	M	M	M	M	M	T	M	T	T	T	T

Amino acids (indicated with the letter code) and their positions (indicated in numbers) in the OsPLDα1 variants. Amino acids with highlighted background show substitutions in OsPLDα1 protein variants in comparison to the OsPLDα1 protein (AB571657.1).

^a^Groups of OsPLDα1 variants based on amino acid sequences.

^b^Groups of *OsPLDα1* variants based on nucleotide sequences.

^c^Ref indicate the nucleotide sequences variants which translates into the amino acid sequence same as that of the reference OsPLDα1 protein sequence of Nipponbare.

### Domains and motifs in OsPLDα1 variants

Domains/motifs were determined and compared in OsPLDα1 protein and its 12 variants. Three important domains including one copy of C2 domain and two copies of Phospholipase D Active site (PLDc) motif were detected in the reference OsPLDα1 protein. C2 domain was found to be present in all the OsPLDα1 variants, however, length of this domain was found 17 amino acids shorter than in VI-XII protein variants. The alignment of C2 domain from reference protein to the variants showed the absence of KFVEGIEDTVGVGKGAT peptide at 36 amino acid position (see Supplementary Fig. S4). In addition to the missing peptide, two amino acid substitutions have also been reported in the C2 domain which included Isoleucine to valine substitution (at position 76) in variant X, XI and XII and asparagine to aspartate substitution (at position 77) in variant X. Further analysis revealed the presence of two copies of PLDc motif in the PLDalpha1 protein (PLDc-I covering 330 to 368 amino acid position and PLDc-II covering 658 to 685 position of amino acids in the PLDalpha1 protein) in all the variants except variant X in which PLDc-I motif was found missing. Alignment of 330 to 368 amino acid sequences from PLDα1 protein to other variants revealed the absence of RIVSFVGGLDLCDGR peptide at amino acid position 354 and eight amino acid substitutions in variant X (see Supplementary Fig. S5).

### Tertiary structure prediction of OsPLDα1 protein in Nipponbare and variant X

**Figure 4:**
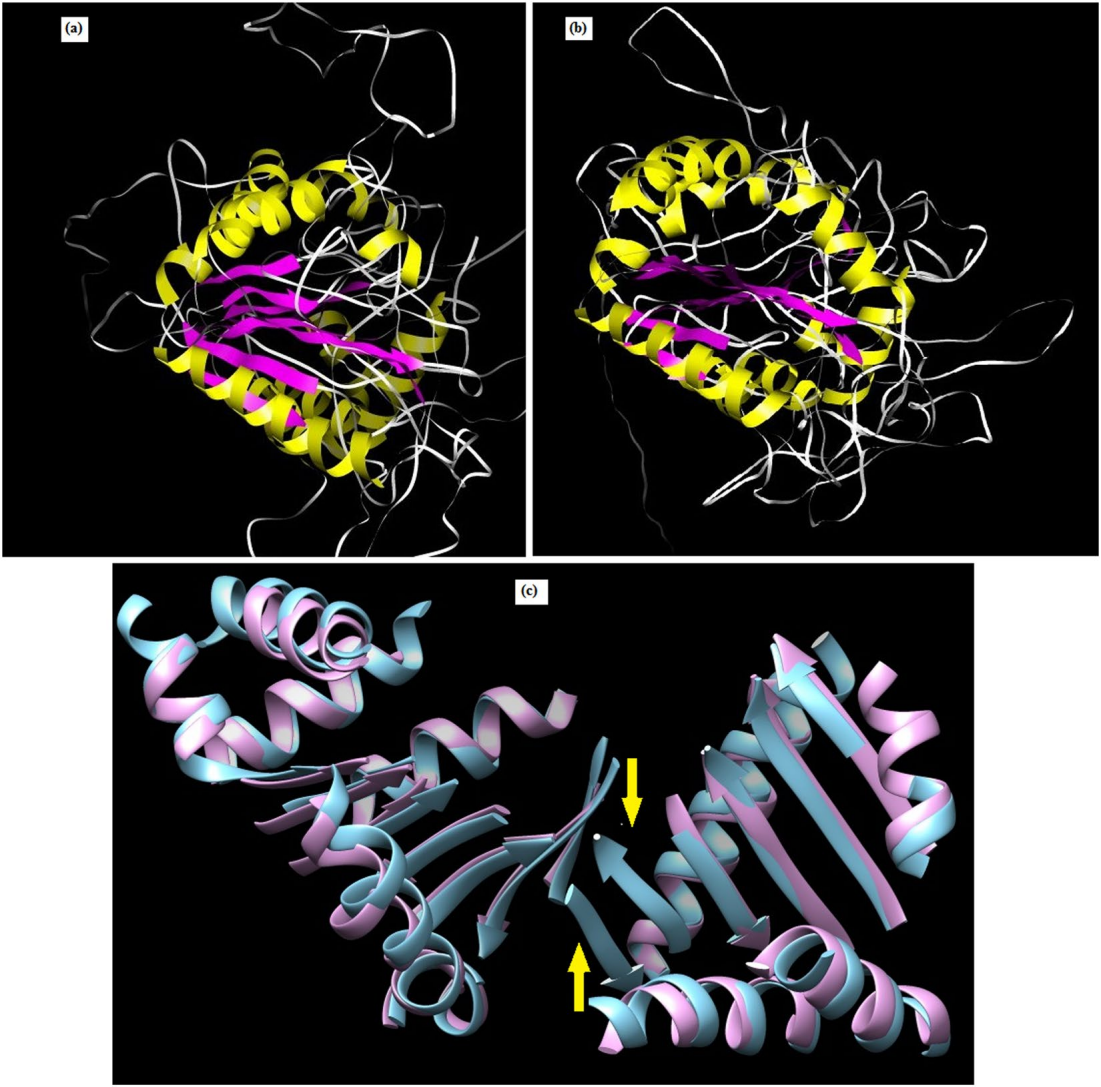
Three-dimensional structures of *OsPLDα1* protein in **(a)** Nipponbare and **(b)** *IR101152* accession of *O. officinalis*. **(c)** Superimposition of *OsPLDα1* protein from Nipponbare and *IR101152* accession of *O. officinalis*. Two β-strands (shown with arrows) were found missing in the *IR101152* (depicted in pink color) upon superimposition with Nipponbare (depicted in blue color).

Homology modeling approach was employed to determine the three-dimensional structures of OsPLDα1 protein from Nipponbare and a representative accession (IR101152 accession *O. officinalis*) of variant X. The MPI Bioinformatics Toolkit (http://toolkit.tuebingen.mpg.de) selected 1v0w as the template for OsPLDα1 protein structure prediction. 1v0w represents the first crystal structure of Phospholipase-D from bacterial source *Streptomyces* sp. strain PMF^35^. By using this template, structures were predicted for OsPLDα1 protein in Nipponbare (Fig. 4a) and IR101152 accession of *O. officinalis* (Fig. 4b). RMSD (Root Mean Square Deviation) values were calculated using chimera and were found to be less than 2 Å (0.745 Å for Nipponbare and 0.825 Å for IR101152) indicating the accuracy of generated structures. Further, the predicted structures were superimposed and results showed the absence of two β-sheets in the IR101152 accession of *O. officinalis* species (Fig. 4c).

### Differential expression of *OsPLDα1* variants

**Figure 5:**
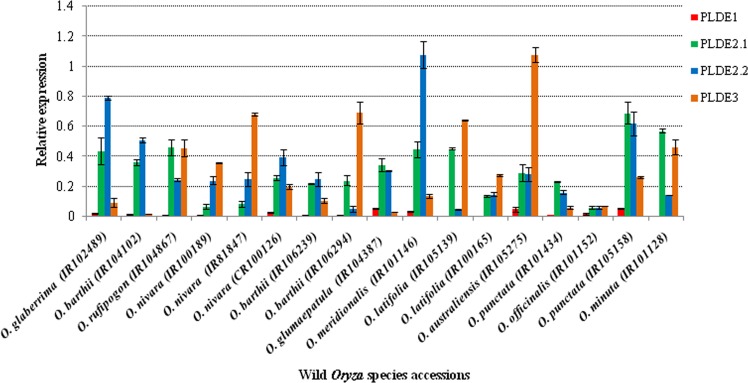
*OsPLDα1* transcript levels in immature seeds from wild *Oryza* species accessions. Mean values for *OsPLDα1* transcripts and standard deviation (S.D.) measured relative to Actin expression. Relative transcript levels of *OsPLDα1*, in accessions of wild *Oryza* species, for four qRT-PCR primers namely PLDE1 (designed from first exon of the gene), PLDE2.1 (designed from 5′end of second exon), PLDE2.2 (designed from 3′end of second exon), and PLDE3 (designed from third exon of the gene) are shown. Among all the wild species accessions, IR101152 accession of *O. officinalis* was found to have lowest transcript levels using all the four qRT-PCR primers.

**Figure 6:**
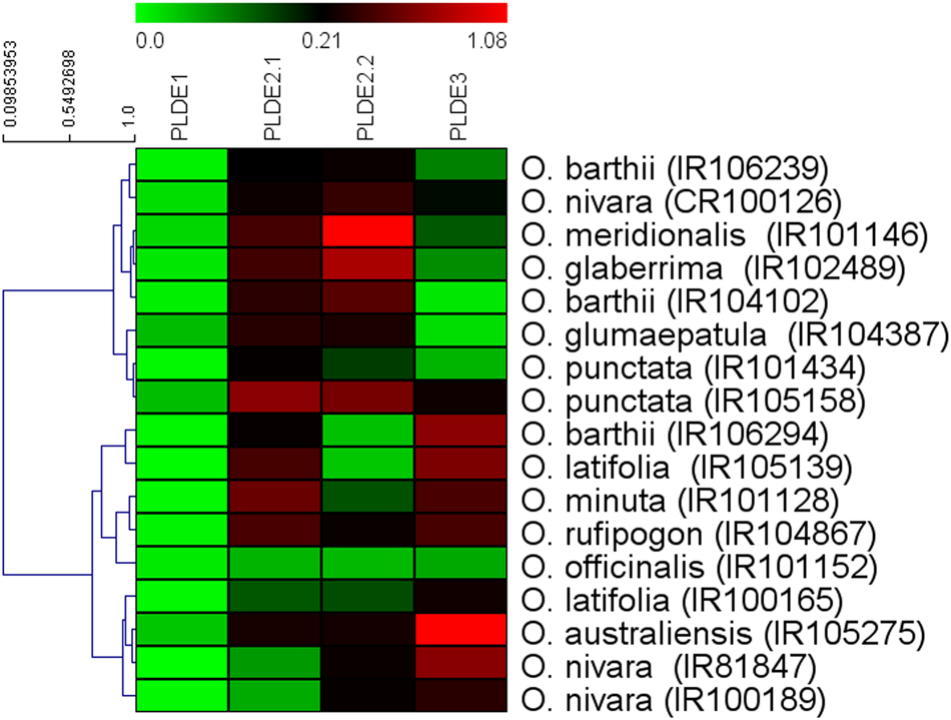
Heatmap showing differential expression of *OsPLDα1* transcripts between as well as within the accessions of wild *Oryza* species. PLDE1, PLDE2.1, PLDE2.2 and PLDE3 denotes the qRT-primers designed from exons of *OsPLDα1* gene. Wild species accessions (horizontal) were hierarchially clustered (Pearson sorrelation, average linkage). Color patterns from green to red indicate low to high transcript levels, thus IRGC101152 have the lowest expression for all the four exon specific qRT primers.

From each of the identified *OsPLDα1* variants, at least one accession was selected for expression profiling. Significant differences were observed for the *OsPLDα1* transcript levels in immature seeds from wild *Oryza* species accessions (Fig. 5). Expression differences for the transcripts acquired with primers designed from 5′ and 3′ ends of the second exon, signified the presence of truncated splice forms in most of the accessions. In addition, expression study also revealed significant expression variations between the genotypes for the same transcript variant and within the same genotype for different transcript variants. Further, for the confirmation of these results, amplification of full length transcript (*Os01t0172400-1*) and two alternate splice forms viz. *Os01t0172400-4* and *Os01t0172400-5* was performed in *Oryza* species accessions. Single sharp bands of expected amplicon size were obtained for all the three transcript forms (see Supplementary Figure S6), which validates that the full length as well as other transcripts with shorter lengths were present in the accessions. Moreover, it varified that the truncations obtained in the accessions are real and not due to failure of cDNA synthesis at the ends of mRNA. Of all the *OsPLDα1* variants, lowest transcript expression (for all the four qRT-primers) was observed in the *O. officinalis* accession (IR101152) followed by *O. punctata* (IR101434). Further, the hierarchical clustering dendrogram represents the *OsPLDα1* transcripts differences between as well as within the wild species accessions (Fig. 6).

## Discussion

The major bottleneck in improving the rice bran quality is narrow genetic base of germplasm on which breeders are working. Hence, the utilization of wild species germplasm to identify the ‘novel alleles’ through sequence based allele mining, and their further transfer to the elite lines has emerged as a good breeding strategy^36^. The progenitor *Oryza* species, in comparison to cultivated rice, are known to carry a number of functionally characterized genes with important coding variations^37^. It leads to the inference that useful coding variations for *OsPLDα1* could be mined from primary and secondary *Oryza* gene pools. The present study depicts an in-depth survey of the genetic variability at *OsPLDα1* in a large panel of genetically and geographically diverse wild rice germplasm. Despite repeated efforts to sequence the second intron in the accessions belonging to genomes other than AA genome, we could not obtain high quality sequence and only multiplets were obtained in that region. The reason for this could be significant sequence differences between the reference *japonica* variety Nipponbare and the species with genomes other than AA genome species. This resulted in fragmented assembly of *OsPLDα1* gene sequence in wild species having CC, EE, BBCC, and CCDD genomes and hence polymorphic and phylogenetic analysis were conducted for individual exons, introns and UTRs to include all the wild species accessions in the study.

### Phylogenetic relationships of *OsPLDα1* gene among diverese wild species germplasm of rice

At exon 1, only two distinguished clusters were observed comprising all the AA genome species in one cluster and other diploid and tetraploid genome species in other cluster (Fig. 1a). The probable explanation for the lower variability is the smaller size of the first exon as compared to other two exons and might have fewer roles in controlling the trait. Among the AA genome species, *O. glaberrima* accessions were found closely related to *O*. *barthii* accessions while *O*. *rufipogon* accessions clustered close to *O*. *sativa* and *O*. *nivara*. It has been already established by earlier studies that African rice *O. glaberrima* was domesticated from the wild progenitor *O. barthii* approximately 3,000 years ago^38^ explaining the relative closeness between the clads. Also, the close genetic relationship between African rice *O. glaberrima* and *O. barthii* has been inferred way back using isozymes as markers^39^ and later by using SSRs and SNPs markers^40,41^. A comparison at major domesticated genes, for instance, *Gn1a* (Grain productivity), *qSH1* (Shattering), *Sd1* (Semi-dwarfing), *Gw2* (Grain width), *GIF1* (Grain incomplete filling), *badh2* (Flavor or fragrance), *Phr1* (Grain discoloration), *OsLG1* (Closed panicle), *Sh4* (Shattering), *Moc1* (Tillering), *Rc* (Red pericarp), *Sdr4* (Seed dormancy), *Ep2* (Erect panicle), *Ipa1* (Ideal plant architecture), *Dep1* (Panicle architecture), and *Sh4* (Shattering) by Wang *et al*.^42^ revealed reduced nucleotide diversity in *O. glaberrima* than *O. barthii* which correlates well with the hypothesis of *O. barthii* being the progenitor harbors greater diversity and during the process of domestication/selection it got reduced in *O. glaberrima*. At two other exons of *OsPLDα1*, *O. rufipogon* and *O. nivara* accessions showed admixture among them as well as with *O. sativa* (*indica* as well as *japonica*) with not much divergence (Fig. 1b,c). This observation could be ascribed to the fact that these two wild species are more closely related and collectively regarded as the progenitors of *O. sativa*^43,44^. Extensive allele sharing between *O. rufipogon* and *O. nivara* has also been documented by Banaticla‐Hilario *et al*.^45^. A taxonomic debate is still continued over whether *O. rufipogon* (the perennial species), *O. nivara* (the annual species), can be considered as two species or ecotypes of the same species^46,47^. Moreover, both *O. rufipogon* and *O. nivara* share a common geographical distribution in South and Southeast Asia therefore the probability of gene flow is higher between them. These species are cross compatible and exhibit little genetic differentiation and is supported by molecular phylogenetic analysis and population studies^48–54^.

At exon 2 and 3, the *O. longistaminata* accessions (distributed in Africa) consistently showed significant differentiation from other AA genome *Oryza* species. This divergence of *O. longistaminata* could be attributed to the unique morphological features such as self-incompatibility, distinctive characteristics of ligules and the presence of rhizomes. These features are not present in any other *Oryza* species which support to the data we obtained^55–57^. Further, haplotype diversity and feature-specific variation has been reported in *O. longistaminata* which the authors attributed to out-crossing nature of this species^58^. The other explanation could be the long distance dispersal of the seeds by animals, birds or any other way followed by ecological differentiation making this species different. The comparative study of genetic relationship using SSR and RAPD markers also revealed clear differentiation of *O. longistaminata* from other AA genome species^59^. Similar observations were also found by other scientists^60–62^. Interestingly, *O. meridinolis* did not group with any of the AA genome cluster. These results are consistent with the findings of evolutionary divergence study at *PSTOL1* locus in wild, domesticated and weedy rice^63^ but contradictory to the findings based on plastome analysis which shows *O. longistaminata* to be most diverged from AA-species^64^.

The present study revealed that the species belonging to the *O. officinalis* complex (*O. officinalis, O. australiensis, O. punctata, O. minuta*, and *O. latifolia*) harbor maximum variability at the *OsPLDα1* locus in comparison to AA genome spp. and *Oryza* cultivars. This complex is the largest one with 10 species and five genome types (BB, CC, EE, BBCC, CCDD) that are distributed widely in Asia, Africa, Australia and Latin America^65^, and hence might be capturing wider variability due to ecological speciation and polyploidization events. It is noteworthy that the *O. officinalis* (CC genome) accessions formed a distinct cluster at second and third exons then the counter diploid species *O. punctata* (BB genome) and two of the tetraploid species *O. minuta* (BBCC) and *O. latifolia* (CCDD) (Fig. 1b,c). These results signify that the *O. officinalis* species carry maximum variability at the *OsPLDα1* locus in comparison to rest of the *Oryza* species. A study on polyploidy evolution in *O. officinalis* complex by Wang *et al*. (2009) states that the CC genome diverges with BB genome at ca.4.8 Mya followed by a series of speciation of C genome diploids and later successive events of polyploidization leads to the formation of tetraploid species *i.e* CCDD at 0.9 Mya and BBCC between ca. 0.3–0.6 Mya^66^. Further, *O. latifolia* (CCDD genome) clustered closer to *O. australiensis* (EE), this can be explained by the fact that EE genome is considered to be progenitor of DD genome^67–69^. Allele mining at *Pi54* locus by Kumari *et al*. (2013) also observed that *O. officinalis, O. punctata* and *O. latifolia* forms a divergent cluster from other AA genome species^70^. A comparison of the sequences of *Xa3/Xa26* orthologous family also revealed very low similarity between cultivated rice and wild *Oryza* species comprising of *O. officinalis* and *O. minuta*^71^.

In addition to the variations in coding region of the *OsPLDα1* gene, the nucleotide changes including large InDels were also detected in the non-coding regions including first and third introns. Contrary to the other genome species, all the AA genome species were found in the same clade as of the Nipponbare, and *O. meridionalis* was found most distantly related of all the AA genome species (Fig. 2a,c). The *Oryza officinalis* complex, in a similar fashion of carrying maximum variability in the coding region, was found to carry maximum variability in the non-coding region as well. These variations included SNPs as well as large InDels (see Supplementary Table S2). The roles of intronic mutations have earlier been found evident in the expressions of *tubulin*, polyubiquitin and waxy (*Wx*) genes of rice^72–74^. The variations detected, in the current study, at the non-coding region of *OsPLDα1* could play significant role in the transcript synthesis and accumulation which might lead to change in trait expression.

### Protein domain analysis in OsPLDα1 variants

In this study, domains and motifs of the Nipponbare OsPLDα1 protein were aligned with the protein sequence of 12 OsPLDα1 variants (see Supplementary Fig. S7). Rice OsPLDα1 contains a single putative C2 domain that has been predicted to be involved in signal transduction and membrane trafficking, and is important in Ca^2+^-regulated binding to phospholipids^75,76^. In plants, Ca^2+^ is an important regulator of PLD activity, C2 domain has been known to play an important role in this regulation^77,78^. In the present study, seven of the identified protein variants viz. VI, VII, VIII, IX, X, XI, and XII were found to have a deletion of 17 amino acid long peptide that also included one of the four conserved amino acids (Glutamic acid at position 42) that are instrumental in Ca^2+^ binding^79^. The variants VIII, IX, X, XI and XII were having a common amino acid substitution at the position 111. In addition, variant X was detected with two unique amino acid substitutions in the C2 domain, at positions 59 and 60. The missing peptide, absence of conserved amino acid, and amino acid substitutions, may further lead to change in the protein function as this domain is important in translocating proteins to memberanes^80,81^. C2 domain deletion mutants in *PI3K* lead to loss of important inter-residue contacts and thereby lead to reduction in binding energy^82^. Downstream of the C2 domain, B-domain was found conserved among all the 12 protein varaints except for a single amino acid substitution in the varaint III. This region is similar to the B-domain of insect and cereal α-amylases that frequently regulate enzyme activity^83–85^.

Each rice PLD consists of two fully conserved HxKxxxxD motifs, which form the active catalytic site for phosphoester bond hydrolysis^86^. Any mutation in the HKD motifs abolishes the enzymatic activity of the PLD enzyme. Our inspection of predicted protein sequences revealed the presence of both of the HKD motifs in all the protein variants (see Supplementary Fig. S7) which is supported by the fact that most eukaryotic PLDs require two functional HKD sites to remain catalytically active^87^. Also, the three amino acid residues involved in PIP_2_ activation were found conserved in all the variants^88^. However, within the first PLD catalytic (PLDc-I) motif of the variant X, 15 amino acid long peptide was missing (see Supplementary Fig. S5) which might also had an altered effect on the enzyme activity.

### *OsPLDα1* gene expression profiling in Wild *Oryza* species and detection of a new *OsPLDα1* transcript

To carry out expression analysis at *OsPLDα1* locus, the plant development stage for RNA extraction was chosen on the basis of expression profiling of 17 PLD isoforms using the expression data from *RiceXProv3.0* database (see Supplementary Fig. S8). The expression analysis using various plant tissues at different developmental stages indicated that the activity of *OsPLDα1* enzyme was very high during early stages of grain development. Moreover, Suzuki (2011) reported the hike of PLD content in the seeds till 3 wk after flowering, becoming stagnant afterwards. Also, no PLD protein band was observed one week after flowering in the seeds of PLD-null rice mutant (03-s108), having <0.01% PLD activity in rice bran when compared to Nipponbare^21^. These results correlate the functional expression of *OsPLDα1* in rice bran and immature seeds. Consequently, for the experiments conducted in the current study, RNA was isolated from immature seeds (one week after flowering). The quantitative gene expression studies have been successfully utilized to study the alterations in the transcript abundance during cell differentiation or development^89^; variation in expression for cells vulnerable to a chemical substance, for instance, drug, toxin, hormone or cytokine)^90^; and as a diagnostic tool^91^.

**Figure 7:**
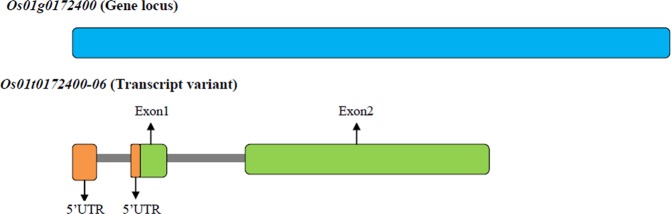
Graphical representation of newly identified *OsPLDα1* transcript variant, *Os01t0172400-06*. A new transcript form having only two exons was detected in the wild *Oryza* species accessions viz. *O. barthii* (IR104102 and IR106239), *O. nivara* (CR100126), *O. glaumaepatula* (IR104387), *O. meridionalis* (IR101146), and *O. punctata* (IR101434 and IR105158); and an accession of *O. glaberrima* (IR102489). Expression profiling in these accessions, using exon specific qRT-PCR primers, showed the low abundance of transcripts having third exon.

For expression analysis, in the present study, four exon-specific qRT-PCR primer pairs were designed from the exonic region of *OsPLDα1* (Table S3). The designed primers aimed to assess the wild genotypes for variability in the gene expression as well as to unveil if the gene is alternatively spliced (see Supplementary Fig. S9). Alternate splicing has been known to control the gene expression and functional diversification of proteins in higher eukaryotes. Alternative splicing of the Ca^2+^-independent phospholipase A_2_ (iPLA_2_) pre-mRNA in humans can result in the production of regulatory subunits that can modify iPLA_2_ *in vivo* activity^92^. Alternative splicing is ubiquitous in rice with 36,650 known splicing events effecting 8772 genes including *OsWRKY62* and *OsWRKY76*^93,94^. Further, differential expression levels of various genes involved in spikelet development in different rice species have been shown to manifest different phenotypes^95^. Our expression profiling results revealed significant differences in the *OsPLDα1* transcript abundance, between the wild *Oryza* species, being lowest in *O. officinalis* spp. followed by *O. punctata* and *O. latifolia* (Fig. 5). In the *O. officinalis* accessions, two insertions viz. A at nucleotide position 459 and T at nucleotide position 1927 led to maximum alterations in the *OsPLDα1* protein that included 14 amino acid substitutions and absence of two peptides (see Supplementary Fig. S4 and S5). The alterations observed in the protein could be the reason for lowest enzymatic activity in *O. officinalis* spp. The novel allele leading to low *OsPLDα1* expression in *O. officinalis* accessions has been named as *OsPLDα1-1a* and is available in NCBI database (http://www.ncbi.nlm.nih.gov) with GenBank accession numbers MF966931, MF966932, and MF966933. In addition, significant differences were observed in the transcript abundance within the accessions for the primers designed from 5′ and 3′ ends of second exon, demonstrating the presence of 5′ and 3′ truncated mRNA (Fig. 5). Interestingly, IR102489 accession of *O. glaberrima* and accessions of wild *Oryza* species including *O. barthii* (IR104102 and IR106239), *O. nivara* (CR100126), *O. glaumaepatula* (IR104387), *O. meridionalis* (IR101146), and *O. punctata* (IR101434 and IR105158) had low abundance of transcripts having third exon when compared to the transcript levels of the first and second exons (Fig. 5). However, the five earlier reported *OsPLDα1* transcript forms confirm the presence of third exon in all the splice forms (see Supplementary Fig. S9). Therefore, the current study revealed the presence of a new *OsPLDα1* transcript variant, named as *Os01t0172400-06*, having truncations before the third exon (Fig. 7).

## Conclusion

The species belonging to *O. officinalis* complex possess maximum variability at the *OsPLDα1* locus. Of the *O. officinalis* complex species, *OsPLDα1* allele of *O. officinalis* accessions has been reported to carry maximum number of non-synonymous SNPs/InDels which further led to alterations in the protein domains, that are responsible for regulating the enzyme activity. The lowest levels of *OsPLDα1* transcript abundance in the *O. officinalis* accessions suggests that the reported polymorphism in the nucleotide and amino acid sequences, varied gene structure, and altered domains play an important role in regulating the enzyme activity in rice bran. Also, a new *OsPLDα1* transcript variant named as *Os01t0172400-06*, having third exon missing in it, was discovered in the present study. We are in the process of transferring the superior *OsPLDα1* allele, identified in *O. officinalis* accession (IRGC101152) i.e., *OsPLDα1-1a* (GenBank accession no. MF966931), into the elite rice cultivars.

## Methods

### Plant materials

A set of 56 accessions including 48 accessions representing 11 wild species of *Oryza* viz. *O. barthii* (n = 6)*, O. nivara* (n = 10)*, O. rufipogon* (n = 16)*, O. longistaminata* (n = 3)*, O. meridionalis* (n = 1)*, O. glumaepatula* (n = 2)*, O. officinalis* (n = 3)*, O. australiensis* (n = 1)*, O. punctata* (n = 2), *O. minuta* (n = 2), and *O. latifolia* (n = 2)]; and 8 accessions of African cultivated rice *O. glaberrima* were undertaken for the current study (Table 1). These germplasm accessions have been actively maintained at Punjab Agricultural University (PAU), Ludhiana, and were originally procured from the International Rice Research Institute (IRRI), Philippines and from National Rice Research Institute (NRRI), Cuttack. The sequence analysis in *Oryza* cultivars namely Punjab Rice 114 (PR 114), Nagina 22 (N22), IR64, Pusa 44, Minghui 63, Feng-Ai-Zhan, and Kitake, revealed the presence of *OsPLDα1* allele. Therefore, these cultivars were used as positive checks to carry out the comparative *OsPLDα1* sequence analysis with wild *Oryza* species accessions. Standard agronomic practices were followed to raise the crop. These practices included sowing of seeds in seedbeds and transplanting one-month-old seedlings in the field with a row-to-row distance of 70 cm and plant-to-plant distance of 45 cm; weed control using a Paddy Weeder, 15 days after transplanting and again after a fortnight; application of organic manures (15 tonnes of farmyard manure per hectare prior to transplanting of rice), bio-fertilizer (treat the nursery seedlings for 45 minutes in the solution made by dissolving 0.5 kg of *Azorhizobium* biofertilizer in requisite amount of water so as to soak seedlings needed to transplant one hectare, and then transplanting was done immediately) along with chemical fertilizers comprising 222 kg/ha Neem coated urea (provide 104 kg/ha Nitrogen), 67 kg/ha Diammonium Phosphate (provide 30 kg/ha phosphorus), and 49 kg/ha Muriate of potash (provide 30 kg/ha potassium) for higher crop yield and maintenance of soil health. 1/3 nitrogen was applied within 2 weeks of transplanting while the whole phosphorus and potassium was applied before the last puddling. Broadcasting of the remaining nitrogen was done in two equal splits, one three weeks after transplanting and the other three weeks afterwards; water was kept standing in the crop continuously for two weeks after transplanting, and thereafter irrigation was done two days after the ponded water has infiltrated into the soil; to facilitate harvesting, irrigation was stopped about a fortnight before maturity; panicles of the plants were covered with the mash bags to avoid shattering of the seeds; harvesting and threshing of different genotypes was done separately to avoid seed admixture.

### DNA extraction, primer designing and PCR amplification

The current study followed a modified CTAB method to isolate genomic DNA from the selected genotypes^96^. 0.8% agarose gel was used to access the quantity and quality of DNA from each sample. For further use, DNA samples were diluted with 1X TE buffer and stored at −20 °C. To PCR amplify the coding and non-coding regions of the *OsPLDα1* variants, *Oryza sativa japonica* cv. Nipponbare sequence (GenBank accession no. AB571657.1) was utilized to design five overlapping primer pairs (Table 4). Supplementary Fig. S10 shows the *OsPLDα1* gene structure (RAPdb ID *Os01g0172400*) and positions of the designed primer pairs along the length of gene. First and last primers were designed from the upstream and downstream flanking regions of the gene to sequence the whole gene. PCR was performed in a 30 μl reaction mix containing 0.3 μl Phusion® high fidelity DNA polymerase, 3 μl of genomic DNA (20ng/μl), 6 μl of 5X HF buffer, 6 μl of dNTPs (1 mM), 3 μl of primer (5 μM), and 11.7 μl Nuclease Free Water. The thermal cycling conditions were as follows: an initial denaturation at 94 °C for 5 min; 35 cycles of 1 min denaturation at 94 °C followed by 1 min annealing at 55 °C and 1 min extension at 72 °C; and a final 5 min extension at 72 °C.

**Table 4:** Overlapping PCR-primer pairs used for amplification of different segments of the *OsPLDα1*.

Primer ID	Forward Primer (5′ to 3′)	Reverse Primer (5′ to 3′)	Amplicon size^a^
PLD-1	TTTAACCTCGCCTCCTCC	TCTCCAATTCTTGTCTACTACC	783 bases (−154–629)
PLD-2	GCCCGAATTTGATCTGCT	TTTGGAATGAAGTTGTCTGG	946 bases (545–1472)
PLD-3	GGAGAGGAGATTGACAGATGG	AGGAGAAGGTGGAATAATAGTG	995 bases (1259–2233)
PLD-4	CATGATATTCACTCACGGCT	TGTAACTCATCTGACATGCT	862 bases (2114–2957)
PLD-5	CTACCTCACTTTCTTCTGCT	ATGTCCCAGTACTTCTCC	885 bases (2749–+16)

^**a**^Numbers in parenthesis shows the position of bases covered by the primer pairs on *OsPLDα1* locus.

### Sequencing of *OsPLDα1* gene in selected accessions and cultivars

Ethidium bromide stained 1.0% agarose gel electrophoresis was performed to analyze PCR products. 1 kb plus ladder (Thermo Scientific Generular) was used to estimate the DNA fragment size. We obtained single sharp bands of expected amplicon size for all the five overlapping primers (see Supplementary Fig. S11). The Wizard® SV PCR Clean-Up System (Promega, USA) as per the manufacturer’s protocol was followed to excise and purify the DNA fragments. The details of targeted DNA nucleotide sequence were created using separate sequencing reactions for forward and reverse primers. The ABI Big-dye Terminator v3.1 chemistry performed the sequencing reaction and ABI Sequencer 3730XL sequenced the DNA fragments, at the School of Agricultural Biotechnology, Punjab Agricultural University, Ludhiana. Experiment was carried out in two replications to confirm the presence of single nucleotide polymorphism (SNPs).

### Analysis of the generated nucleotide sequences and protein prediction

For comparative sequence analysis, DNA Baser v4.23.0 (http://www.dnabaser.com/) software joined the contigs produced by overlapping primers, and generated the consensus sequence of *OsPLDα1* gene. This software also helps in automatic identification and clipping of poor quality regions at both ends of the sequences. ClustalX 2.1.1 software was undertaken to generate the multiple sequence alignment^97^. *OsPLDα1* sequence from ‘Nipponbare’ rice variety, which contains normal levels of PLD activity i.e., 133.2 units/mg^21^, was used as reference (wild type) in this study. The identified SNPs and InDels were then manually curated by comparing chromatogram files to the ClustalX alignment files.

HMM-based FGENESH online program (http://www.softberry.com/berry.phtml?topic=fgenesh) was used to predict the gene structure and amino acid sequences in different genotypes which were further compared with the Nipponbare protein to detect amino acid substitutions and InDels. Pfam (http://pfam.xfam.org/) online program predicted the domains and motifs in the protein variants. Bioinformatics toolkit (http://toolkit.tuebingen.mpg.de/) was used to predict the tertiary structures of protein. The MODELLER Homology modeling approach was followed^98^ to determine the structure of proteins based on the known strucure of template protein. Ramachandran plots were developed using Procheck through PDBsum (http://www.ebi.ac.uk/thornton-srv/databases/pdbsum) to check the quality of protein models. UCSF Chimera helped to visualize and compare the modeled protein structures^99^. All the developed tertiary structures were superimposed to detect the structural variations. Uniprot (http://www.uniprot.org/uniprot/P84147) online program determined the catalytic sites in the protein.

### Phylogenetic analysis

The MEGA7 software^100^ was used to generate the phylogenetic tree using multiple sequence alignment file. The evolutionary distances were computed using the Maximum Composite Likelihood method with 1,000 bootstrap and are in the units of the number of base substitutions per site.

### RNA isolation, cDNA synthesis, and expression analysis using qRT-PCR

To collect the RNA sample at the same stage (one week after flowering, at milking stage of grain development) from different wild species and cultivars, flowering data was collected on the daily basis. For each genotype, tissue for RNA isolation was collected in a way that each experimental replicate represents RNA from three biological replic ates^101^.

The TRIzol® reagent (Thermo Fisher Scientific) was used for RNA isolation  according to the manufacturer’s protocol. The expression analysis part of the study wa s done at the School of Biology and Ecology, University of Maine, Orono, USA. NanoDrop® ND-1000 estimated the RNA quantity for the different samples. We employed an iScript cDNA kit (Bio-Rad laboratories, CA, USA) which produces first strand cDNAs by reverse transcribing RNA. Sequences of *OsPLDα1* loci and its transcript forms (Locus ID *Os01g0172400*), for qRT primers designing, were retrieved from RAP data base (http://rapdb.dna.affrc.go.jp/viewer/gbrowse/).

Using the Primer-BLAST tool (http://www.ncbi.nlm.nih.gov/tools/primer-blast/), four exon-specific qRT-primer pairs (PLDE1, PLDE2.1, PLDE2.2, and PLDE3) (see Supplementary Table S3) were designed from the exonic regions of *OsPLDα1*. qRT-primers were generated to assess the wild genotypes for variations in abundance of *OsPLDα1* transcript variants (see Supplementary Fig. S9). Each primer was dissolved in 1X TE buffer (stock solution) to have a master stock of 100 µM. Real-time PCR was performed in MyiQ^™^ thermal cycler (Bio-Rad Laboratories, CA, USA) using the iQ^™^ SYBR^®^ Green Supermix (Bio-Rad) according to the manufacturers protocol. The cycling conditions were as follows: 95 °C for 30s, 40 cycles of 95 °C for 5s and 60 °C for 30s. Each sample was amplified in triplicate to confirm the results. 2^−∆CT^ method was used to calculate the relative expression levels^102^, and the *actin* (Locus ID *Os10g0510000*) gene was used as an internal control to normalize the data. For validation of expression results, primers were designed for the full length amplification of three alternate splice forms viz., *Os01t0172400-1*, *Os01t0172400-4* and *Os01t0172400-5* (see Supplementary Table S4).

## Supplementary information

Supplementary.

## Data Availability

The DNA sequences of wild species accessions and cultivars generated and analyzed during the current study are available in the GenBank repository with accession numbers: *O. glaberrima* [IRGC100854 (**MF774061**), IRGC101800 (**MF919319**), IRGC102196 (**MF919320**), IRGC102489 (**MF919321**), IRGC102512 (**MF919322**), IRGC102600b (**MF919323**), IRGC102925 (**MF919324**), IRGC103750 (**MF919325**)]; *O. bartii* [IRGC100117 (**MF919326**), IRGC101317 (**MF919327**), IRGC104102 (**MF919328**), IRGC105990 (**MF919329**), IRGC106239 (**MF919330**), IRGC106294 (**MF919331)**]; *O. nivara* [CR100008 (**MF919334**), CR100400 (**MF919335**), CR100429 (**MF91336**), IRGC80547 (**MF919332**), IRGC81847 (**MG725633**), IRGC92713 (**MF919337**), IRGC92930 (**MG725634**), IRGC100189 (**MF919338**), IRGC106397 (**MF919333**)]; *O. rufipogon* [CR100013 (**MG725637**), IRGC80610 (**MG725635**), IRGC81976 (**MG725638**), IRGC83823 (**MG725640**), IRGC89224 (**MG725641**), IRGC99551 (**MG725642**), IRGC103308 (**MF919339**), IRGC104308 (**MG725643**) IRGC104867 (**MF966922**), IRGC105491 (**MG7256360**), IRGC105569 (**MF966923**), IRGC105902 (**MF966924**), IRGC106162 (**MF966925**), IRGC106336 (**MF966926**), IRGC106433 (**MF966927**), IRGC113652 (**MF966928**)]; *O. longistaminata* [IRGC101200 (**MG725645**), IRGC104301 (**MG725646**), IRGC105206 (**MG725647**)]; *O. meridionalis* [IRGC101146 (**MG725648**)]; *O. glumaepatula* [IRGC100184 (**MF966930**), IRGC104387 (**MF966929**)]; *O. officinalis* [IRGC101152 (**MF966931**), IRGC105674 (**MF966932**), IRGC106501 (**MF966933**)]; *O. australiensis* [IRGC105275 (**MG725650**)]; *O. punctata* [IRGC105158 (**MG725651**)]; *O. minuta* [IRGC101100 (**MG725652**), IRGC101128 (**MG725653**)]; *O. latifolia* [IRGC105139 (**MG725654**)]; Feng-Ai-Zhan (**MF975521**); Kitake (**MF975522**); Minghui63 (**MF975523**); Nagina22 (**MF975524**); PR114 (**MF975525**); Pusa44 (**MF975526**).
