# Supplementary material for: Novel allelic variation in the Phospholipase D alpha1 gene (OsPLDα1) of wild Oryza species implies to its low expression in rice bran
Source: Sci Rep. 2020 Apr 20;10:6571. doi: 10.1038/s41598-020-62649-w (PMC7170842; doi:10.1038/s41598-020-62649-w)
Supplement: Supplementary file 1 — Supplementary. [file 41598_2020_62649_MOESM1_ESM.pdf]

# Novel allelic variation in the *Phospholipase D alpha1* gene (*OsPLDα1*) of wild *Oryza* species implies to its low expression in rice bran

Amandeep Kaur[1,2], Kumari Neelam[1], Karminderbir Kaur[1], Ai Kitazumi[2,#a], Benildo G. de los Reyes[2,#a] and Kuldeep Singh[1,#b*]

[1] *School of Agricultural Biotechnology, Punjab Agricultural University, Ludhiana, Punjab, India*

[2] *School of Biology and Ecology, University of Maine, Orono, Maine, United States of America*

[#a] *Current address: Department of Plant and Soil Science, Texas Tech University, Lubbock, Texas, United States of America*

[#b] *Current address: ICAR- National Bureau of Plant Genetic Resources, New Delhi, India*

*Corresponding author: Kuldeep Singh; Email: (kuldeep.singh4@icar.gov.in)

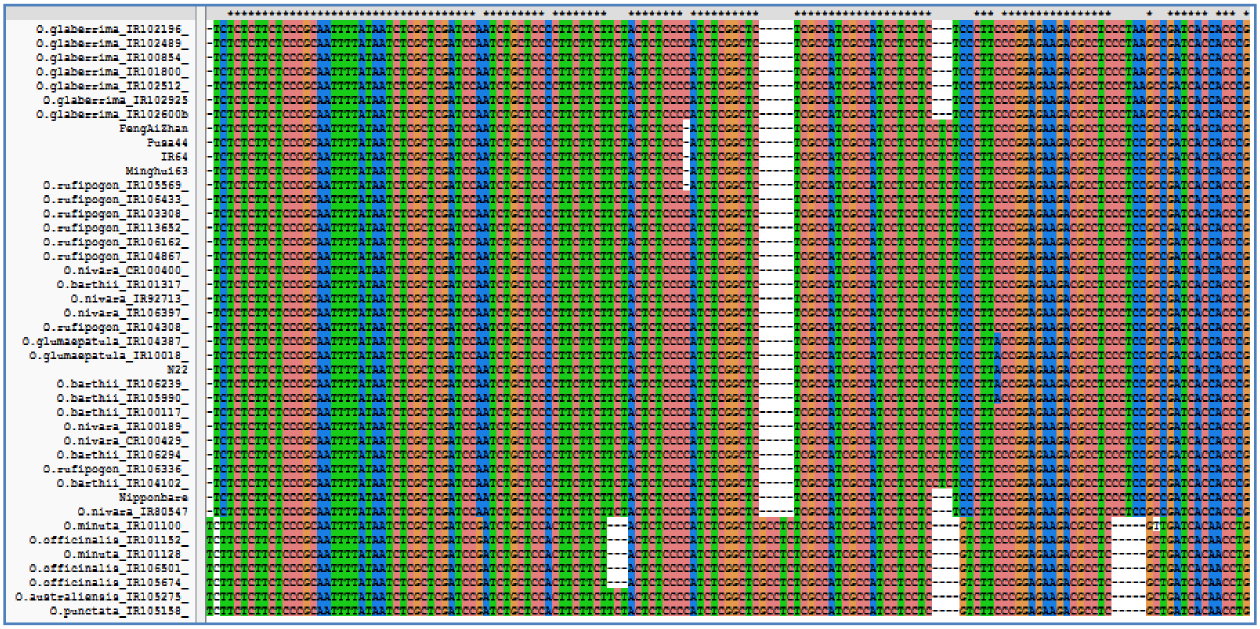

**Figure S1. Alignment of first Untranslated Region (UTR1) of the *OsPLDα1* gene across wild species accessions and *Oryza* cultivars.** It shows the presence of SNPs and InDels in the *O. officinalis* complex and AA genome wild *Oryza* species as well. Deletion of a nucleotide C at position 69 in the *Oryza* cultivars is also shown.

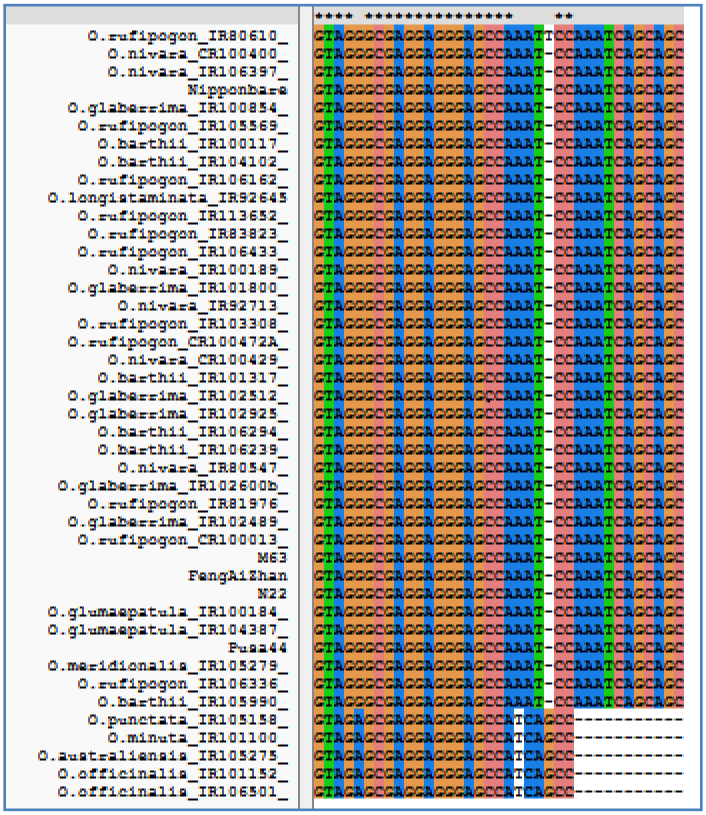


**Figure S2. Alignment of second Untranslated Region (UTR2) of the *OsPLDα1* gene across wild species accessions and *Oryza* cultivars.** It shows the presence of SNPs and InDels in the species belonging to *O. officinalis* complex.

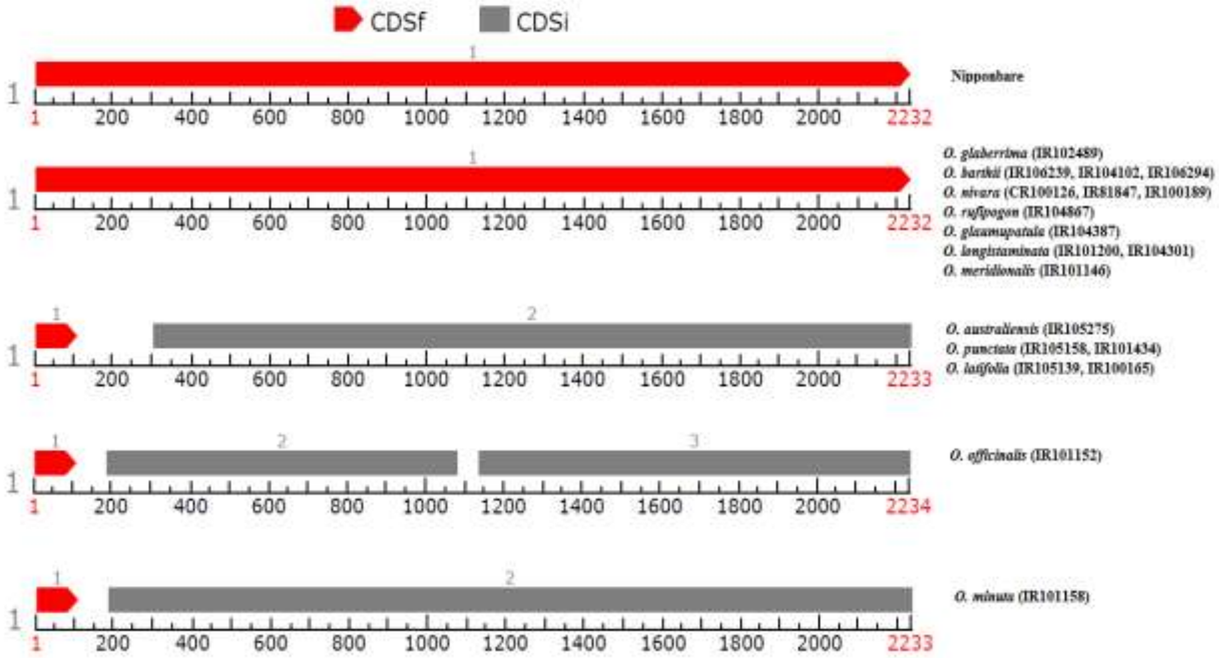


**Figure S3. Comparison of *OsPLDα1* cDNA structure in Nipponbare and wild *Oryza* species accessions.** HMM-based FGENESH online program was used to predict the *OsPLDα1* cDNA isoforms in wild *Oryza* species. The alignment revealed altered *OsPLDα1* cDNA in the wild *Oryza* species belonging to *O. officinalis* complex, due to alterations in the nucleotide sequence of exon2 while exon1 and exon 3 were of same length as of the Nipponbare. At the end of first exon, a gap of 193bp (from nucleotide position 109 to 301) was detected in *O. australiensis, O. punctata, and O. latifolia* accessions while a smaller gap of 77 bp (from nucleotide position 109 to 184) was detected in the *O. minuta* and *O. officinalis* accessions. *O. officinalis* accession had an additional gap within the exon2 from the nucleotide position 1081 to 1137.

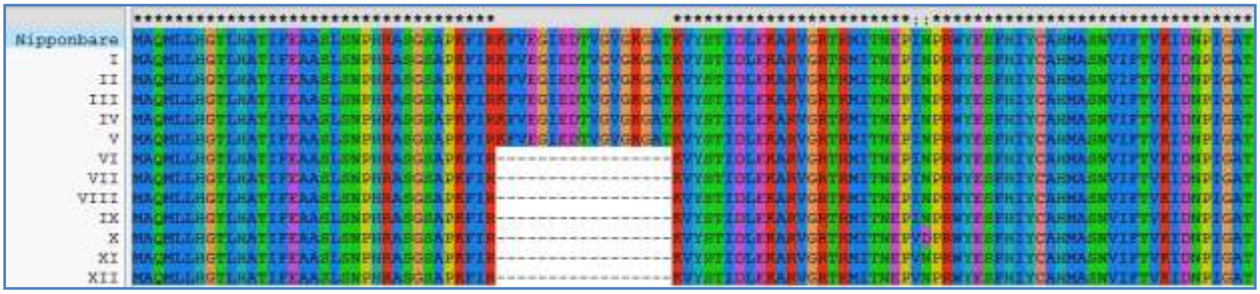


**Figure S4. Alignment of C2-domain across 12 OsPLDα1 protein variants.** Alignment of the C2-domain shows the amino acid substitution and a deletion of peptide (KFVEGIEDTVGVGKGAT), at position 29, in the protein variants VI-XIII.

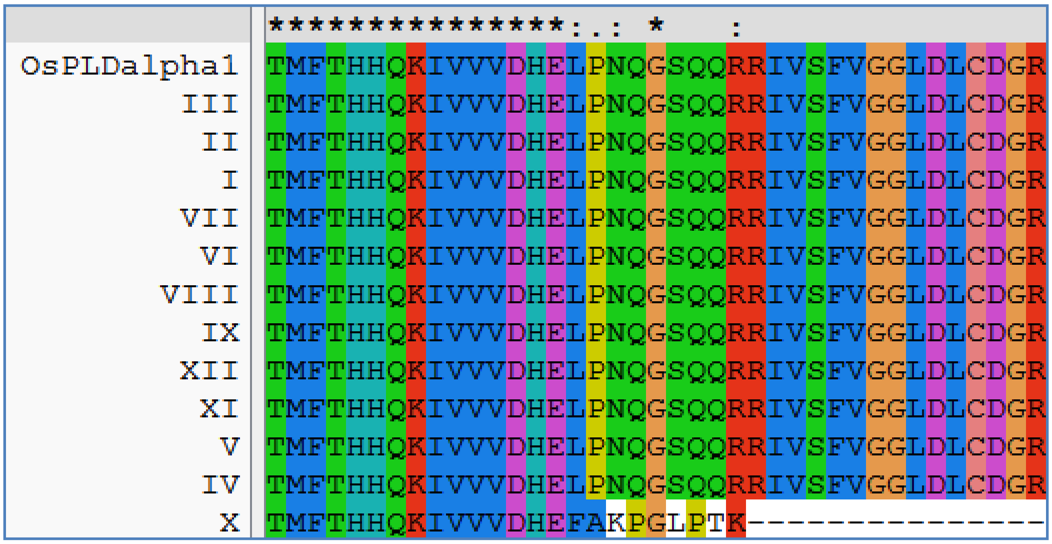


**Figure S5. Alignment of PLDcI-domain across 12 OsPLDα1 protein variants.** Alignment of the PLDc-I domain (330-368) shows the absence of RIVSFVGGLDLCDGR peptide at amino acid position 354 and eight amino acid substitutions in variant X.

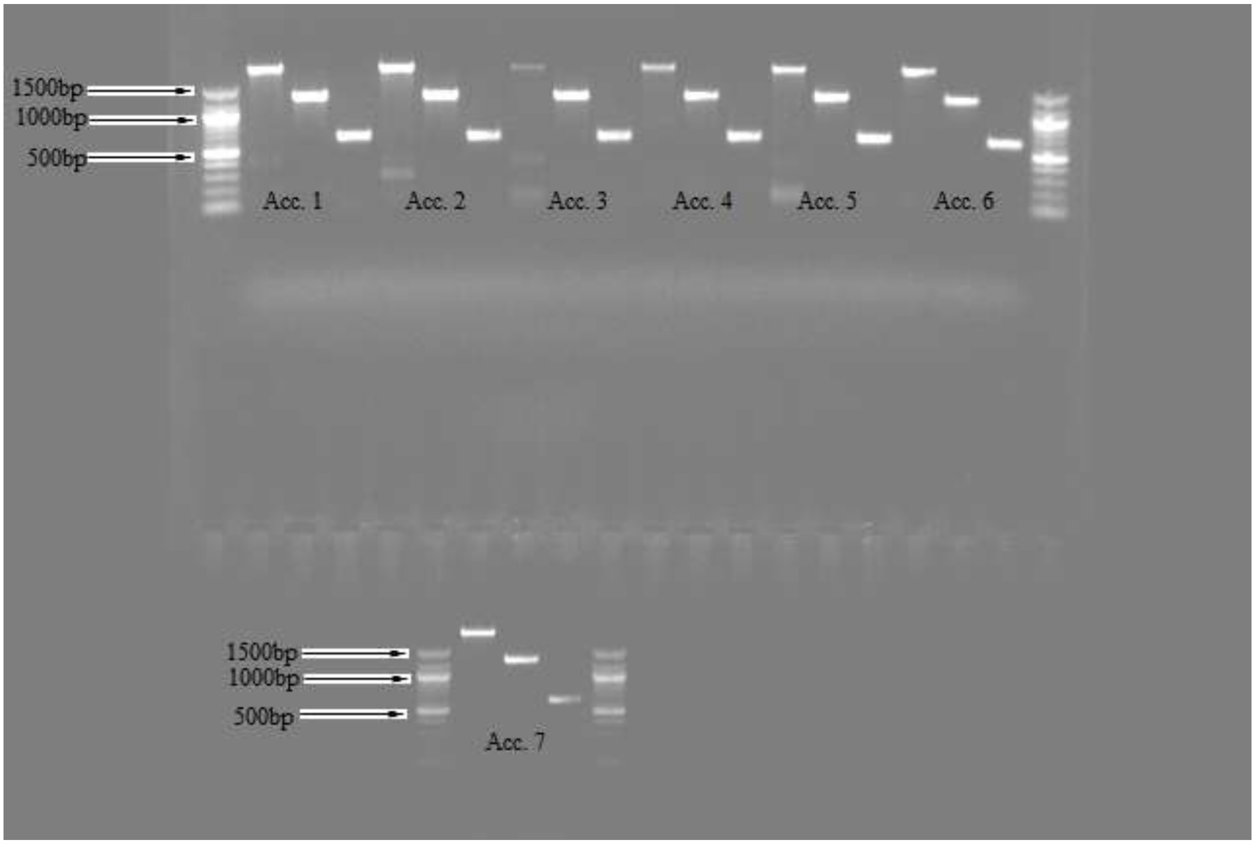


**Figure S6. Amplification of full length *OsPLDα1* transcript variants in the accessions belonging to different *Oryza* species**. Three amplicons within an accession denote the amplification of different sized *PLDalpha1* transcripts i.e. *Os0t1072400-1* (2248bp), *Os0t1072400-4* (1280bp), and *Os0t1072400-5* (622bp). Acc. 1 to Acc. 7 represents accessions of different *Oryza* species viz. *O. glaberrima* (IRGC102489), *O. meridionalis* (IRGC101146), *O. latifolia* (IRGC101165), *O. officinalis* (IRGC101152), *O. punctata* (IRGC105158), *O. minuta* (IRGC101128), and *O. australiensis* (IRGC105275), respectively.

```
                        C2-Domain
Nipponbare   MAQMLLHGTLHATIFEAASLSNPHRASGSAPKFIRKFVEGIEDTVGVGKGATKVYSTIDL  60
I            MAQMLLHGTLHATIFEAASLSNPHRASGSAPKFIRKFVEGIEDTVGVGKGATKVYSTIDL  60
II           MAQMLLHGTLHATIFEAASLSNPHRASGSAPKFIRKFVEGIEDTVGVGKGATKVYSTIDL  60
III          MAQMLLHGTLHATIFEAASLSNPHRASGSAPKFIRKFVEGIEDTVGVGKGATKVYSTIDL  60
IV           MAQMLLHGTLHATIFEAASLSNPHRASGSAPKFIRKFVEGIEDTVGVGKGATKVYSTIDL  60
V            MAQMLLHGTLHATIFEAASLSNPHRASGSAPKFIRKFVEGIEDTVGVGKGATKVYSTIDL  60
VI           MAQMLLHGTLHATIFEAASLSNPHRASGSAPKFIR-----------------KVYSTIDL  43
VII          MAQMLLHGTLHATIFEAASLSNPHRASGSAPKFIR-----------------KVYSTIDL  43
VIII         MAQMLLHGTLHATIFEAASLSNPHRASGSAPKFIR-----------------KVYSTIDL  43
IX           MAQMLLHGTLHATIFEAASLSNPHRASGSAPKFIR-----------------KVYSTIDL  43
X            MAQMLLHGTLHATIFEAASLSNPHRASGSAPKFIR-----------------KVYSTIDL  43
XI           MAQMLLHGTLHATIFEAASLSNPHRASGSAPKFIR-----------------KVYSTIDL  43
XII          MAQMLLHGTLHATIFEAASLSNPHRASGSAPKFIR-----------------KVYSTIDL  43
             ***********************************                 ********

Nipponbare   EKARVGRTRMITNEPINPRWYESFHIYCAHMASNVIFTVKIDNPIGATNIGRAYLPVQEL  120
I            EKARVGRTRMITNEPINPRWYESFHIYCAHMASNVIFTVKIDNPIGATNIGRAYLPVQEL  120
II           EKARVGRTRMITNEPINPRWYESFHIYCAHMASNVIFTVKIDNPIGATNIGRAYLPVQEL  120
III          EKARVGRTRMITNEPINPRWYESFHIYCAHMASNVIFTVKIDNPIGATNIGRAYLPVQEL  120
IV           EKARVGRTRMITNEPINPRWYESFHIYCAHMASNVIFTVKIDNPIGATNIGRAYLPVQEL  120
V            EKARVGRTRMITNEPINPRWYESFHIYCAHMASNVIFTVKIDNPIGATNIGRAYLPVQEL  120
VI           EKARVGRTRMITNEPINPRWYESFHIYCAHMASNVIFTVKIDNPIGATNIGRAYLPVQEL  103
VII          EKARVGRTRMITNEPINPRWYESFHIYCAHMASNVIFTVKIDNPIGATNIGRAYLPVQEL  103
VIII         EKARVGRTRMITNEPINPRWYESFHIYCAHMASNVIFTVKIDNPIGATNIGRAYLPVQEL  103
IX           EKARVGRTRMITNEPINPRWYESFHIYCAHMASNVIFTVKIDNPIGATNIGRAYLPVQEL  103
X            EKARVGRTRMITNEPVDPRWYESFHIYCAHMASNVIFTVKIDNPIGATNIGRAYLPVQEL  103
XI           EKARVGRTRMITNEPVNPRWYESFHIYCAHMASNVIFTVKIDNPIGATNIGRAYLPVQEL  103
XII          EKARVGRTRMITNEPVNPRWYESFHIYCAHMASNVIFTVKIDNPIGATNIGRAYLPVQEL  103
             ***************::*******************************************
                                                          B-Domain
Nipponbare   LNGEEIDRWLDICDNNREPVGESKIHVKLQYFDVSKDRNWARGVRSTKYPGVPYTFFSQR  180
I            LNGEEIDRWLDICDNNREPVGESKIHVKLQYFDVSKDRNWARGVRSTKYPGVPYTFFSQR  180
II           LNGEEIDRWLDICDNNREPVGESKIHVKLQYFDVSKDRNWARGVHSTKYPGVPYTFFSQR  180
III          LNGEEIDRWLDICDNNREPVGESKIHVKLQYFDVSKDRNWARGVRSTKYPGVPYTFFSQR  180
IV           LNGEEIDRWLDICDNNREPVGESKIHVKLQYFDVSKDRNWARGVRSTKYPGVPYTFFSQR  180
V            LNGEEIDRWLDICDNNREPVGESKIHVKLQYFDVSKDRNWARGVRSTKYPGVPYTFFSQR  180
VI           LNGEEIDRWLDICDNNREPVGESKIHVKLQYFDVSKDRNWARGVRSTKYPGVPYTFFSQR  163
VII          LNGEEIDRWLDICDNNREPVGESKIHVKLQYFDVSKDRNWARGVRSTKYPGVPYTFFSQR  163
VIII         LNGEEIDTWLDICDNNREPVGESKIHVKLQYFDVSKDRKWARGVRSTKYPGVPYTFFSQR  163
IX           LNGEEIDTWLDICDNNREPVGESKIHVKLQYFDVSKDRNWARGVRSTKYPGVPYTFFSQR  163
X            LNGEEIDTWLDICDNNREPVGESKIHVKLQYFDVSKDRNWARGVRSTKYPGVPYTFFSQR  163
XI           LNGEEIDTWLDICDNNRELVGESKIHVKLQYFDVSKDRNWARGVRSTKYPGVPYTFFSQR  163
XII          LNGEEIDTWLDICDNNREPVGESKIHVKLQYFDVSKDRNWARGVRSTKYPGVPYTFFSQR  163
             ******* ********** *******************:*****:***************

Nipponbare   QGCKVTLYQDAHVPDNFIPKIPLADGKNYEPHRCWEDIFDAISNAQHLIYITGWSVYTEI  240
I            QGCKVTLYQDAHVPDNFIPKIPLADGKNYEPHRCWEDIFDAISNAQHLIYITGWSVYTEI  240
II           QGCKVTLYQDAHVPDNFIPKIPLADGKNYEPHRCWEDIFDAISNAQHLIYITGWSVYTEI  240
III          QGCKVTLYQDAHVPDNFIPKIPLADGKNYEPHRCWEDIFDAISNAQHLIYITGWSVYTKI  240
IV           QGCKVTLYQDAHVPDNFIPKIPLADGKNYEPHRCWEDIFDAISNAQHLIYITGWSVYTEI  240
V            QGCKVTLYQDAHVPDNFIPKIPLADGKNYEPHRCWEDIFDAISNAQHLIYITGWSVYTEI  240
VI           QGCKVTLYQDAHVPDNFIPKIPLADGKNYEPHRCWEDIFDAISNAQHLIYITGWSVYTEI  223
VII          QGCKVTLYQDAHVPDNFIPKIPLADGKNYEPHRCWEDIFDAISNAQHLIYITGWSVYTEI  223
VIII         QGCKVTLYQDAHVPDNFIPKIPLADGKNYEPHRCWEDIFDAISNAQHLIYITGWSVYTEI  223
IX           QGCKVTLYQDAHVPDNFIPKIPLADGKNYEPHRCWEDIFDAISNAQHLIYITGWSVYTEI  223
X            QGCKVTLYQDAHVPDNFIPKIPLADGKNYEPHRCWEDIFDAISNAQHLIYITGWSVYTEI  223
XI           QGCKVTLYQDAHVPDNFIPKIPLADGKNYEPHRCWEDIFDAISNAQHLIYITGWSVYTEI  223
XII          QGCKVTLYQDAHVPDNFIPKIPLADGKNYEPHRCWEDIFDAISNAQHLIYITGWSVYTEI  223
             **********************************************************:*

Nipponbare   TLVRDSNRPKPGGDVTLGELLKKKASEGVRVLMLVWDDRTSVGLLKRDGLMATHDEETEN  300
I            TLVRDSNRPKPGGDVTLGELLKKKASEGVRVLMLVWDDRTSVGLLKRDGLMATHDEETEN  300
II           TLVRDSNRPKPGGDVTLGELLKKKASEGVRVLMLVWDDRTSVGLLKRDGLMATHDEETEN  300
III          TLVRDSNRPKPGGDVTLGELLKKKASEGVRVLMLVWDDRTSVGLLKRDGLMATHDEETEN  300
IV           TLVRDSNRPKPGGDVTLGELLKKKASEGVRVLMLVWDDRTSVGLLKRDGLMATHDEETEN  300
V            TLVRDSNRPKPGGDVTLGELLKKKASEGVRVLMLVWDDRTSVGLLKRDGLMATHDEETEN  300
VI           TLVRDSNRPKPGGDVTLGELLKKKASEGVRVLMLVWDDRTSVGLLKRDGLMATHDEETEN  283
VII          TLVRDSNRPKPGGDVTLGELLKKKASEGVRVLMLVWDDRTSVGLLKRDGLMATHDEETEN  283
VIII         TLIRDSNRPKPGGDVTLGELLKKKASEGVRVLMLVWDDRTSVGLLKRDGLMATHDEETAN  283
IX           TLVRDSNRPKPGGDVTLGELLKKKASEGVRVLMLVWDDRTSVGLLKRDGLMATHDEETAN  283
X            TLVRDSNRPKPGGDVTLGELLKKKASEGVRVLMLVWDDRTSVGLLKRDGLMATHDEETAN  283
XI           TLVRDSNRPKPGGDVTLGELLKKKASEGVRVLMLVWDDRTSVGLLKRDGLMATHDEETAN  283
XII          TLVRDSNRPKPGGDVTLGELLKKKASEGVRVLMLVWDDRTSVGLLKRDGLMATHDEETAN  283
             **:*******************************************************  *
```

```
                                                     HxKxxxxD Motif-1 [PLDc-I]
Nipponbare   YFHGSDVNCVLCPRNPDDSGSIVQDLSISTMFTHHQKIVVVDHELPNQGSQQRRIVSFVG 360
I            YFHGSDVNCVLCPRNPDDSGSIVQDLSISTMFTHHQKIVVVDHELPNQGSQQRRIVSFVG 360
II           YFHGSDVNCVLCPRNPDDSGSIVQDLSISTMFTHHQKIVVVDHELPNQGSQQRRIVSFVG 360
III          YFHGSDVNCVLCPRNPDDSGSIVQDLSISTMFTHHQKIVVVDHELPNQGSQQRRIVSFVG 360
IV           YFHGSNVNCVLCPRNPDDSGSIVQDLSISTMFTHHQKIVVVDHELPNQGSQQRRIVSFVG 360
V            YFHGSDVNCVLCPRNPDDSGSIVQDLSISTMFTHHQKIVVVDHELPNQGSQQRRIVSFVG 360
VI           YFHGSDVNCVLCPRNPDDSGSIVQDLSISTMFTHHQKIVVVDHELPNQGSQQRRIVSFVG 343
VII          YFHGSDVNCVLCPRNPDDSGSIVQDLSISTMFTHHQKIVVVDHELPNQGSQQRRIVSFVG 343
VIII         YFHGSDVNCVLCPRNPDDSSSIVQDLSISTMFTHHQKIVVVDHELPNQGSQQRRIVSFVG 343
IX           YFHGSDVNCVLCPRNPDDSGSIVQDLSISTMFTHHQKIVVVDHELPNQGSQQRRIVSFVG 343
X            YFHGSDVNCVLCPRNPDDSGSIVQDLSISTMFTHHQKIVVVDHEFAKPGLPTK------- 336
XI           YFHGSDVNCVLCPRNPDDSGSIVQDLSISTMFTHHQKIVVVDHELPNQGSQQRRIVSFVG 343
XII          YFHGSDVNCVLCPRNPDDSGSIVQDLSISTMFTHHQKIVVVDHELPNQGSQQRRIVSFVG 343
             *****:**************.***********************:.: *   :
                                                 PIP2 Binding
Nipponbare   GLDLCDGRYDTQYHSLFRTLDSTHHDDFHQPNFATASIKKGGPREPWHDIHSRLEGPIAW 420
I            GLDLCDGRYDTQYHSLFRTLDSTHHDDFHQPNFATASINKGGPREPWHDIHSRLEGPIAW 420
II           GLDLCDGRYDTQYHSLFRTLDSTHHDDFHQPNFATASIKKGGPREPWHDIHSRLEGPIAW 420
III          GLDLCDGRYDTQYHSLFRTLDSTHHDDFHQPNFATASIKKGGPREPWHDIHSRLEGPIAW 420
IV           GLDLCDGRYDTQYHSLFRTLDSTHHDDFHQPNFATASIKKGGPREPWHDIHSRLEGPIAW 420
V            GLDLCDGRYDTQYHSLFRTLDSTHHDDFHQPNFANASIKKGGPREPWHDIHSRLEGPIAW 420
VI           GLDLCDGRYDTQYHSLFRTLDSTHHDDFHQPNFANASIKKGGPREPWHDIHSRLEGPIAW 403
VII          GLDLCDGRYDTQYHSLFRTLDSTHHDDFHQPNFANASIKKGGPREPWHDIHSRLEGPIAW 403
VIII         GLDLCDGRYDTQYHSLFRTLDSTHHDDFHQPNFANASITKGGPREPWHDIHSRLEGPIAW 403
IX           GLDLCDGRYDTQYHSLFRTLDSTHHDDFHQPNFANASIKKGGPREPWHDIHSRLEGPIAW 403
X            -----------EDSQLRWTLDSTHHDDFHQPNFANASITKGGPREPWHDIHSRLEGPIAW 385
XI           GLDLCDGRYDTQYHSLFRTLDSTHHDDFHQPNFANASITKGGPREPWHDIHSRLEGPIAW 403
XII          GLDLCDGRYDTQYHSLFRTLDSTHHDDFHQPNFANASITKGGPREPWHDIHSRLEGPIAW 403
                        :  .*  ****************.***.*********************

Nipponbare   DVLYNFEQRWRKQGGKDLLLQLRDLSDTIIPPSPVMFPEDRETWNVQLFRSIDGGAAFGF 480
I            DVLYNFEQRWRKQGGKDLLLQLRDLSDTIIPPSPVMFPEDRETWNVQLFRSIDGGAAFGF 480
II           DVLYNFEQRWRKQGGKDLLLQLRDLSDTIIPPSPVMFPEDRETWNVQLFRSIDGGAAFGF 480
III          DVLYNFEQRWRKQGGKDLLLQLRDLSDTIIPPSPVMFPEDRETWNVQLFRSIDGGAAFGF 480
IV           DVLYNFEQRWRKQGGKDLLLQLRDLSDTIIPPSPVMFPEDRETWNVQLFRSIDGGAAFGF 480
V            DVLYNFEQRWRKQGGKDLLLQLRDLSDTIIPPSPVMFPEDRETWNVQLFRSIDGGAAFGF 480
VI           DVLYNFEQRWRKQGGKDLLLQLRDLSDTIIPPSPVMFPEDRETWNVQLFRSIDGGAAFGF 463
VII          DVLYNFEQRWRKQGGKDLLLQLRDLSDTIIPPSPVMFPEDRETWNVQLFRSIDGGAAFGF 463
VIII         DVLYNFEQRWRKQGGKDLLLQLRDLSDTIIPPSPVMFPEDRETWNVQLFRSIDGGAAFGF 463
IX           DVLYNFEQRWRKQGGKDLLLQLRDLSDTIIPPSPVMFPEDRETWNVQLFRSIDGGAAFGF 463
X            DVLYNFEQRWRKQGGKDLLLQLRDLSDTIIPPSPVMFPEDRETWNVQLFRSIDGGAAFGF 445
XI           DVLYNFEQRWRKQGGKDLLLQLRDLSDTIIPPSPVMFPEDRETWNVQLFRSIDGGAAFGF 463
XII          DVLYNFEQRWRKQGGKDLLLQLRDLSDTIIPPSPVMFPEDRETWNVQLFRSIDGGAAFGF 463
             ************************************************************

Nipponbare   PDTPEEAAKAGLVSGKDQIIDRSIQDAYIHAIRRAKNFIYIENQYFLGSSYAWKPEGIKP 540
I            PDTPEEAAKAGLVSGKDQIIDRSIQDAYIHAIRRAKNFIYIENQYFLGSSYAWKPEGIKP 540
II           PDTPEEAAKAGLVSGKDQIIDRSIQDAYIHAIRRAKNFIYIENQYFLGSSYAWKPEGIKP 540
III          PDTPEEAAKAGLVSGKDQIIDRSIQDAYIHAIRRAKNFIYIENQYFLGSSYAWKPEGIKP 540
IV           PDTPEEAAKAGLVSGKDQIIDRSIQDAYIHAIRRAKNFIYIENQYFLGSSYAWKPEGIKP 540
V            PDTPEEAAKAGLVSGKDQIIDRSIQDAYIHAIRRAKNFIYIENQYFLGSSYAWKPEGIKP 540
VI           PDTPEEAAKAGLVSGKDQIIDRSIQDAYIHAIRRAKNFIYIENQYFLGSSYAWKPEGIKP 523
VII          PDTPEEAAKAGLVSGKDQIIDRSIQDAYIHAIRRAKNFIYIENQYFLGSSYAWKPEGIKP 523
VIII         LDTPEEAAKAGLVSGKDQIIDRSIQDAYIHAIRRAKNFIYIENQYFLGSSYAWKPEGIKP 523
IX           PDTPEEAAKAGLVSGKDQIIDRSIQDAYIHAIRRAKNFIYIENQYFLGSSYAWKPEGIKP 523
X            PDTPEEAAKAGLVSGKDQIIDRSIQDAYIHAIRRAKNFIYIENQYFLGSSYAWKPEGIKP 505
XI           PDTPEEAAKAGLVSGKDQIIDRSIQDAYIHAIRRAKNFIYIENQYFLGSSYAWKPEGIKP 523
XII          PDTPEEAAKAGLVSGKDQIIDRSIQDAYIHAIRRAKNFIYIENQYFLGSSYAWKPEGIKP 523
             ************************************************************

Nipponbare   EDIGALHLIPKELALKVVSKIEAGERFTVYVVVPMWPEGVPESGSVQAILDWQRRTMEMM 600
I            EDIGALHLIPKELALKVVSKIEAGERFTVYVVVPMWPEGVPESGSVQAILDWQRRTMEMM 600
II           EDIGALHLIPKELALKVVSKIEAGERFTVYVVVPMWPEGVPESGSVQAILDWQRRTMEMM 600
III          EDIGALHLIPKELALKVVSKIEAGERFTVYVVVPMWPEGVPESGSVQAILDWQRRTMEMM 600
IV           EDIGALHLIPKELALKVVSKIEAGERFTVYVVVPMWPEGVPESGSVQAILDWQRRTMEMM 600
V            EDIGALHLIPKELALKVVSKIEAGERFTVYVVVPMWPEGVPESGSVQAILDWQRRTMEMM 600
VI           EDIGALHLIPKELALKVVSKIEAGERFTVYVVVPMWPEGVPESGSVQAILDWQRRTMEMM 583
VII          EDIGALHLIPKELALKVVSKIEAGERFTVYVVVPMWPEGVPESGSVQAILDWQRRTMEMM 583
VIII         EDIGALHLIPKELALKVVSKIEAGERFTVYVVVPMWPEGVPESGSVQAILDWQRRTMEMM 583
IX           EDIGALHLIPKELALKVVSKIEAGERFTVYVVVPMWPEGVPESGSVQAILDWQRRTMEMM 583
X            EDIGALHLIPKELALKVVSKIEAGERFTVYVVVPMWPEGVPESGSVQAILDWQRRTMEMM 565
XI           EDIGALHLIPKELALKVVSKIEAGERFTVYVVVPMWPEGVPESGSVQAILDWQRRTMEMM 583
XII          EDIGALHLIPKELALKVVSKIEAGERFTVYVVVPMWPEGVPESGSVQAILDWQRRTMEMM 583
             ************************************************************
```

```
Nipponbare      YTDITEALQAKGIEANPKDYLTFFCLGNREVKQAGEYQPEEQPEADTDYSRAQEARRFMI 660
I               YTDITEALQAKGIEANPKDYLTFFCLGNREVKQAGEYQPEEQPEADTDYSRAQEARRFMI 660
II              YTDITEALQAKGIEANPKDYLTFFCLGNREVKQAGEYQPEEQPEADTDYSRAQEARRFMI 660
III             YTDITEALQAKGIEANPKDYLTFFCLGNREVKQAGEYQPEEQPEADTDYSRAQEARRFMI 660
IV              YTDITEALQAKGIEANPKDYLTFFCLGNREVKQAGEYQPEEQPEADTDYSRAQEARRFMI 660
V               YTDITEALQAKGIEANPKDYLTFFCLGNREVKQAGEYQPEEQPEADTDYSRAQEARRFMI 660
VI              YTDITEALQAKGIEANPKDYLTFFCLGNREVKQGGEYQPEEQPEADTDYSRAQEARRFMI 643
VII             YTDITEALQAKGIEANPKDYLTFFCLGNREVKQAGEYQPEEQPEADTDYSRAQEARRFMI 643
VIII            YTDITEALQAKGIEANPKDYLTFFCLGNREVKQGGEYQPEEQPEADTDYSRAQEARRFMI 643
IX              YTDITEALQAKGIEANPKDYLTFFCLGNREVKQGGEYQPEEQPEADTDYSRAQEARRFMI 643
X               YTDITEALQAKGIEANPKDYLTFFCLGNREVKQGGEYQPEEQPEADTDYSRAQEARRFMI 625
XI              YTDITEALQAKGIEANPKDYLTFFCLGNREVKQAGEYQPEEQPEADTDYSRAQEARRFMI 643
XII             YTDITEALQAKGIEANPKDYLTFFCLGNREVKQAGEYQPEEQPEADTDYSRAQEARRFMI 643
                *********************************.**************************

                  HxKxxxxD Motif-2 [PLDc-II]
Nipponbare      YVHTKMMIVDDEYIIIGSANINQRSMDGARDSEIAMGGYQPYHLATRQPARGQIHGFRMA 720
I               YVHTKMMIVDDEYIIIGSANINQRSMDGARDSEIAMGGYQPYHLATRQPARGQIHGFRMA 720
II              YVHTKMMIVDDEYIIIGSANINQRSMDGARDSEIAMGGYQPYHLATRQPARGQIHGFRMA 720
III             YVHTKMMIVDDEYIIIGSANINQRSMDGARDSEIAMGGYQPYHLATRQPARGQIHGFRMA 720
IV              YVHTKMMIVDDEYIIIGSANINQRSMDGARDSEIAMGGYQPYHLATRQPARGQIHGFRMA 720
V               YVHTKMMIVDDEYIIIGSANINQRSMDGARDSEIAMGGYQPYHLATRQPARGQIHGFRMA 720
VI              YVHTKMMIVDDEYIIIGSANINQRSMDGARDSEIAMGGYQPYHLATRQPARGQIHGFRMA 703
VII             YVHTKMMIVDDEYIIIGSANINQRSMDGARDSEIAMGGYQPYHLATRQPARGQIHGFRMA 703
VIII            YVHTKMMIVDDEYIIIGSANINQRSMDGARDSEIAMGGYQPYHLATRQPARGQIHGFRMA 703
IX              YVHTKMMIVDDEYIIIGSANINQRSMDGARDSEIAMGGYQPYHLATRQPARGQIHGFRMA 703
X               YVHTKMMIVDDEYIIIGSANINQRSMDGARDSEIAMGGYQPYHLATRQPARGQIHGFRMA 685
XI              YVHTKMMIVDDEYIIIGSANINQRSMDGARDSEIAMGGYQPYHLATRQPARGQIHGFRMA 703
XII             YVHTKMMIVDDEYIIIGSANINQRSMDGARDSEIAMGGYQPYHLATRQPARGQIHGFRMA 703
                ************************************************************

Nipponbare      LWYEHLGMLDDVFQRPESLECVQK 744
I               LWYEHLGMLDDVFQRPESLECVQK 744
II              LWYEHLGMLDDVFQRPESLECVQK 744
III             LWYEHLGMLDDVFQRPESLECVQK 744
IV              LWYEHLGMLDDVFQRPESLECVQK 744
V               LWYEHLGMLDDVFQRPESLECVQK 744
VI              LWYEHLGMLDDVFQRPESLECVQK 727
VII             LWYEHLGTLDDVFQRPESLECVQK 727
VIII            LWYEHLGMLDDVFQRPESLECVQK 727
IX              LWYEHLGTLDDVFQRPESLECVQK 727
X               LWYEHLGTLDDVFQRPESLECVQK 709
XI              LWYEHLGTLDDVFQRPESLECVQK 727
XII             LWYEHLGTLDDVFQRPESLECVQK 727
                ******* ****************
```

**Figure S7. Amino acid sequence alignments of the 12 OsPLDα1 variants with the reference protein sequence of Nipponbare**. Amino acids having grey highlights denote the amino acid substitutions in the variants when compared to the reference. The underlined amino acid sequence shows the position of C2-domain. The bold-faced residues indicate the positions of $Ca^{2+}$ binding acidic amino acids. Two HxKxxxxD phosphatidyl transferase motifs are *underlined/italics.* The basic residues in the consensus PIP2-binding motif are bold and underlined. B-Domain is *bold/italics*. Dashes (–) indicate the missing amino acids.

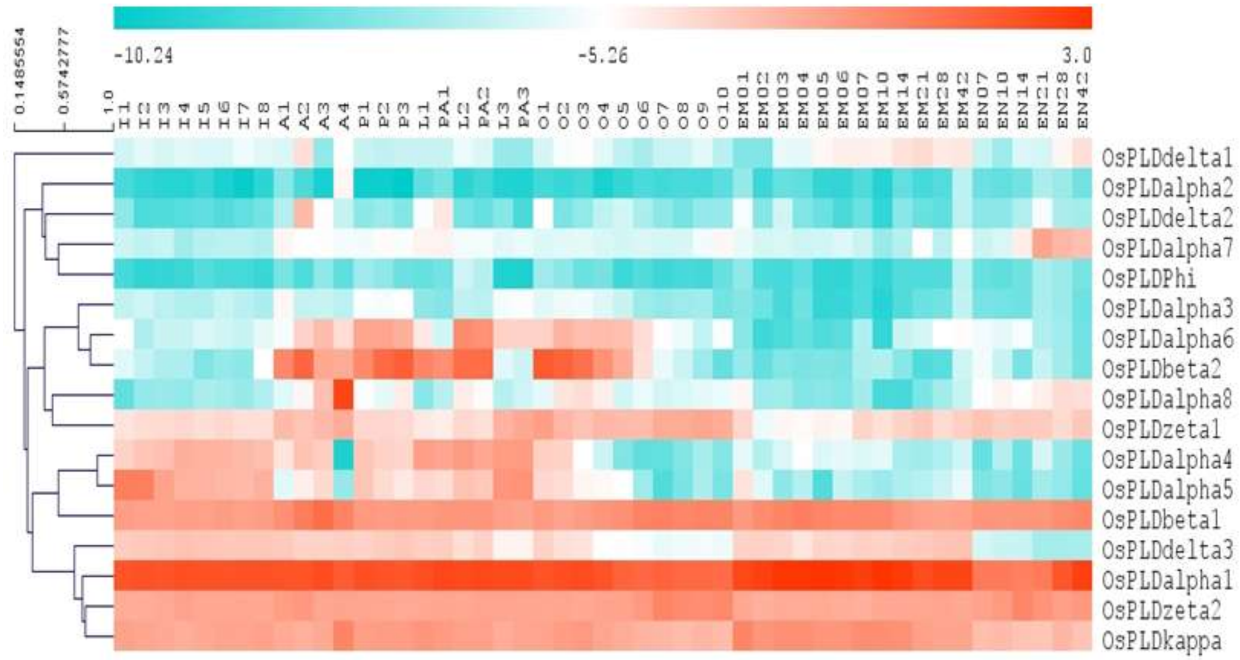


**Figure S8. Hierarchical clustering dendrogram showing highest expression of *OsPLDalpha1*, among 17 isoforms within PLD family, at all the stages of grain development**. Developmental stages comprising eight stages of infloresence [ I1(0.6-1.0 mm), I2 (1.0-1.5mm), I3 (1.5-2.0mm), I4 (2.0-2.5mm), I5 (2.5-3.0mm), I6 (3.0-4.0mm), I7 (4.0-5.0mm), I8 (5.0-10mm)]; four stages of anther [(A1 (0.3-0.6mm), A2 (0.7-1.0mm), A3 (1.2-1.5mm), A4 (1.6 2.0mm); three stages of pistil [(1.5-2.0 cm panicle, 10-14 cm panicle, 14-18 cm panicle)]; three stages of lemma and palea each [L1(1.5-2.0 mm floret), PA1 ((1.5-2.0 mm floret), L2 (4.0-5.0 mm floret), PA2 (4.0-5.0 mm floret), L3 (7 mm floret), PA (7mm floret); ten stages of ovary development [O1 (one day after flowering) to O10 (ten days after floweing ); twelve stages of embryo development [EM01 (one day after flowering) to EM42 (fourty two days after flowring)]; six stages of endosperm (EN) [07 (seven days after flowering ) to 42 (fourty two days after flowering)]. The color scale at the top of the heat map is given in log2 intensity value.

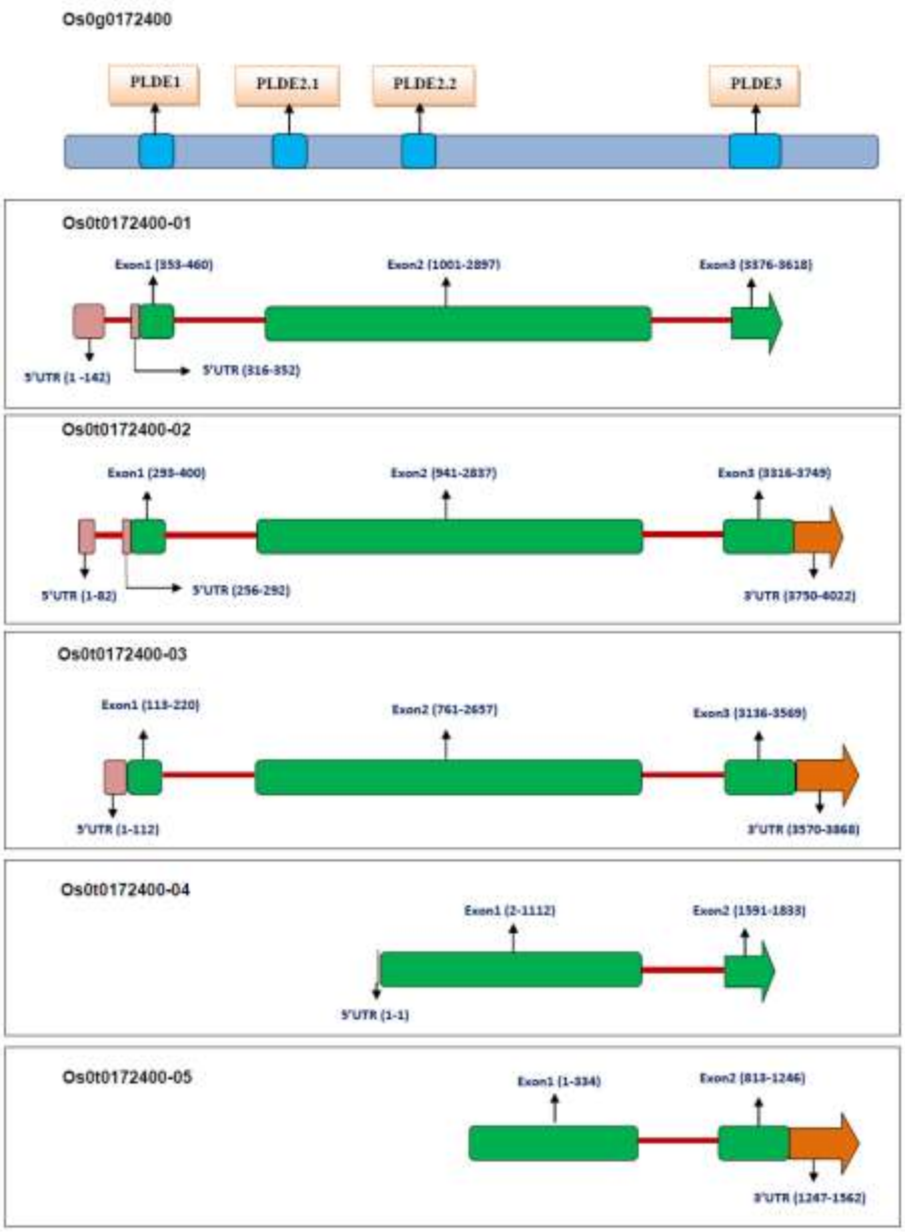


**Figure S9. Five transcipt variants of Os*PLDα1* gene.** It shows the position of the four qRT-PCR primers (PLDE1, PLDE2.1, PLDE2.2, and PLDE3) which were designed from different exons, in a way to evaluate variations in relative abundance of different splice forms.

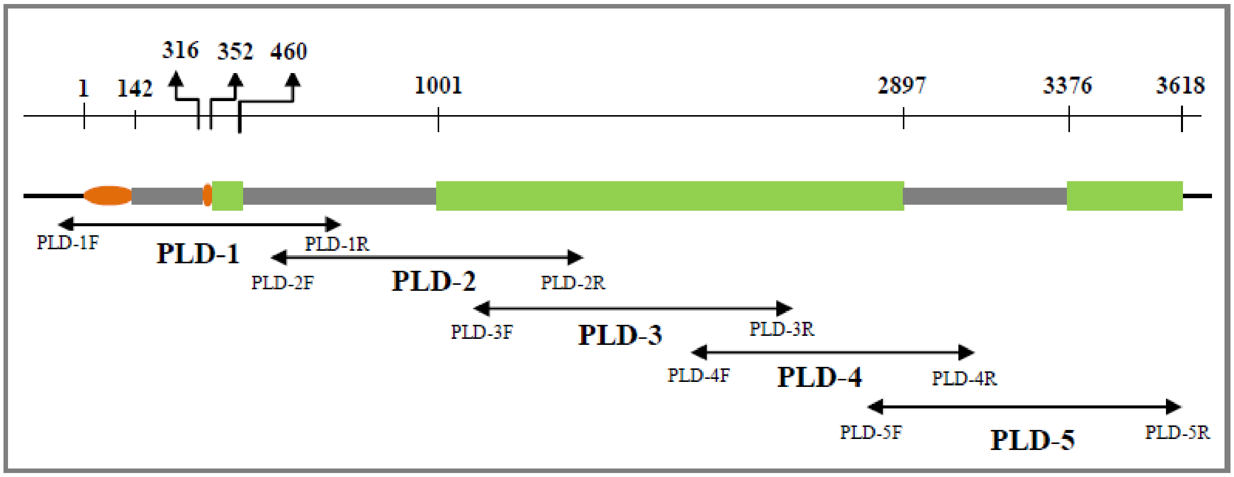


**Figure S10. Schematic representation of gene structure of *OsPLDα1* in Nipponbare.** Positions covered by five overlapping PCR-primers [PLD1 (-154bp to 629bp), PLD2 (545bp to 1472bp), PLD3 (1259bp to 2233bp), PLD4 (2114bp to 2957bp), and PLD5 (2749bp to +16bp)], designed to amplify and sequence the targeted gene are shown. Forward primer of PLD1 and reverse primer of PLD5 were designed from the upstream and downstram flanking regions of the gene, respectively. Sequences of the designed PCR-primer pairs are PLD-1F (TTTAACCTCGCCTCCTCC), PLD-1R (TCTCCAATTCTTGTCTACTACC), PLD-2F (GCCCGAATTTGATCTGCT), PLD-2R (TTTGGAATGAAGTTGTCTGG), PLD-3F (GGAGAGGAGATTGACAGATGG), PLD-3R (AGGAGAAGGTGGAATAATAGTG), PLD-4F (CATGATATTCACTCACGGCT), PLD-4R (TGTAACTCATCTGACATGCT), PLD-5F (CTACCTCACTTTCTTCTGCT), and PLD-5R (ATGTCCCAGTACTTCTCC). Orange color in the figure depicts the UTRs, Green color depicts exons, and Gray color shows the intronic positions. Scale shows the relative positions of UTRs, exons and introns at the *OsPLDα1* locus.

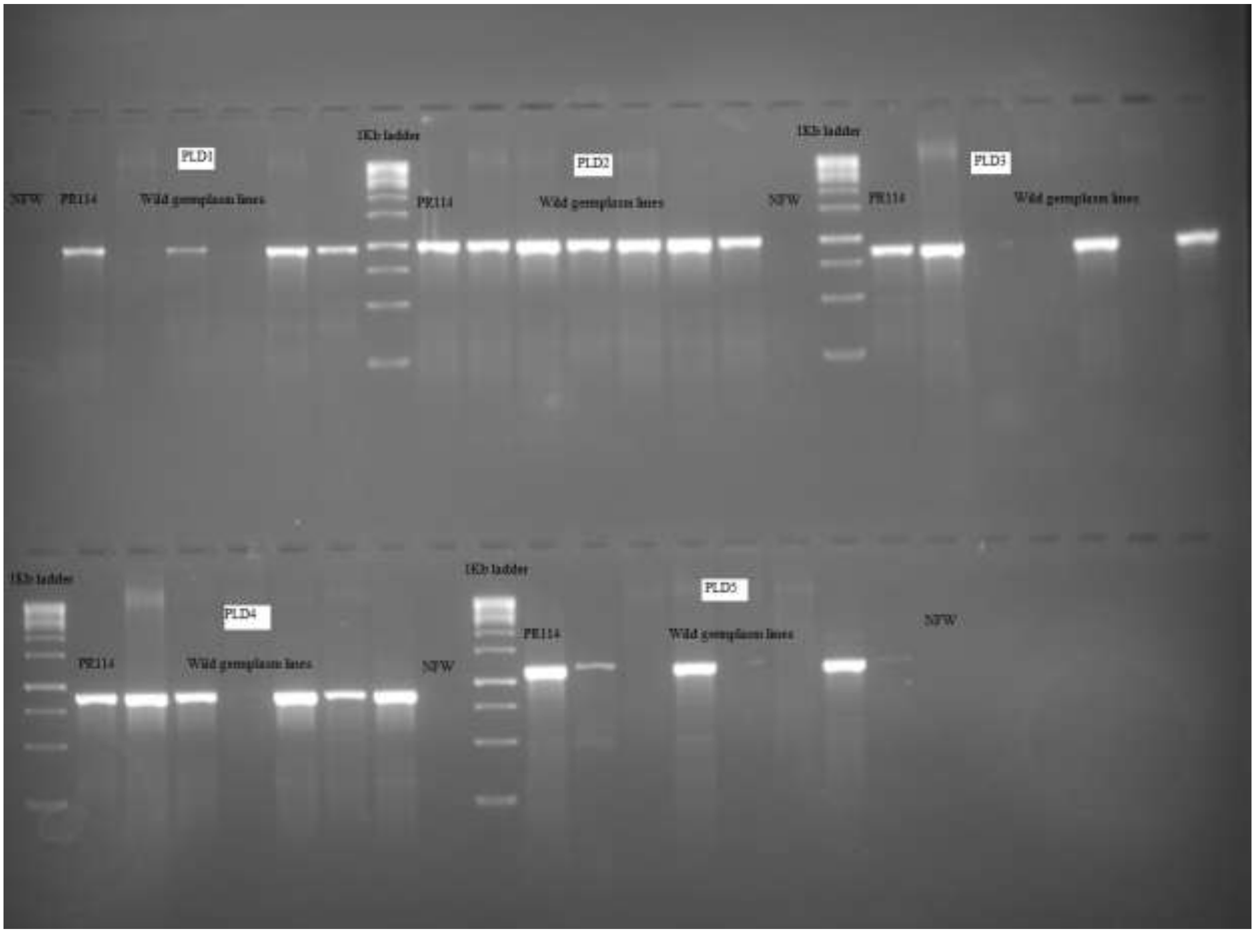


**Figure S11. Single sharp DNA bands of expected amplicon size were obtained using five overlapping primers designed at the *OsPLDα1* locus.** PLD1, PLD2, PLD3, PLD4, and PLD5 denote the five overlapping primers. Wild germplasm lines represent random accessions selected from different wild *Oryza* species. Nuclease Free Water (NFW) was used as a negative control while PR114, a cultivar of rice, has been used as the positive control. Missing amplicons for a few accessions denotes the low quantity and/or low quality of DNA. 1kb plus ladder from Thermo-Scientific has been used to estimate the size of amplicons.

**Table S1**. Nucleotide changes detected within the coding region of *OsPLDα1* across the wild *Oryza* species accessions and rice cultivars in comparison to Nipponbare reference sequence.

| Nucleotide changes | *Oryza* wild species accessions/cultivars |
|---|---|
| **Exon1** | |
| T373C; **+A$_{459}$** | *O. officinalis* (IR101152, IR105674, IR106501); *O. australiensis* (IR105275); *O. punctata* (IR101434, IR105158); *O. minuta* (IR101100, IR101128); *O. latifolia* (IR100165, IR105139) |
| **Exon 2** | |
| G1027A; G1043A; A1118G; C1234T | *O. officinalis* (IR101152, IR105674, IR106501); *O. punctata* (IR105158); *O. minuta* (IR101100, IR101128) |
| T1060C; C1123T; T1162C; C1273T; G1275C; A1788C | *O. officinalis* (IR101152, IR105674, IR106501); *O. australiensis* (IR105275); *O. punctata* (IR101434, IR105158); *O. minuta* (IR101100, IR101128) |
| T1084C | *O. australiensis* (IR105275); *O. punctata* (IR101434) |
| A1087T; C1120T; A1121G; C1387T; T1723C; A2081G; **+T$_{1927}$** | *O. officinalis* (IR101152, IR105674, IR106501) |
| A1087C | *O. australiensis* (IR105275); *O. punctata* (IR101434, IR105158); *O. minuta* (IR101100, IR101128) |
| G1090C; T1258C; C1450G; T1760C; T2048C; G2675A | *O. punctata* (IR101434) |
| T1135C | *O. meridionalis* (IR101146); *O. officinalis* (IR101152, IR105674, IR106501); *O. punctata* (IR105158); *O. minuta* (IR101100, IR101128) |
| G1141A | *O. latifolia* (IR100165, IR105139); *O. barthii* (IR106239, IR106294, IR 105990); *O. nivara* (IR92713, IR92930, IR106397, CR100400, CR100429, CR100126); *O. rufipogon* (IR80610, IR83823, IR99551, IR104308, IR105491, IR105569); *O. glumaepatula* (IR104387); Pusa44, Feng-Ai-Zhan, Minghui63, PR114, IR64, N22 |
| T1153C; T1207C | *O. meridionalis* (IR101146); *O. officinalis* (IR101152, IR105674, IR106501); *O. australiensis* (IR105275); *O. punctata* (IR101434, IR105158); *O. minuta* (IR101100, IR101128) |
| C1156T | *O. meridionalis* (IR101146); *O. australiensis* (IR105275); *O. punctata* (IR101434) |
| T1168A; C1967T; G2108A | *O. meridionalis* (IR101146) |
| G1210A; G1386A | *O. nivara* (IR81847) |
| C1282T; A1962G; C2087A | *O. longistaminata* (IR101200) |
| C1303T; A1825G | *O. officinalis* (IR101152, IR105674, IR106501); *O. punctata* (IR101434, IR105158); *O. minuta* (IR101100, IR101128) |
| C1308T | *O. punctata* (IR105158) |
| C1348T | *O. officinalis* (IR101152, IR105674, IR106501); *O. minuta* (IR101100, IR101128) |
| T1369A; G1619A; G1850A; T1858C; G1870A; C2335T | *O. australiensis* (IR105275) |
| C1516T; G1753A; T1964C; T2102A; T2258C; A2297C; | *O. officinalis* (IR101152, IR105674, IR106501); *O. australiensis* (IR105275); *O. punctata* (IR101434, IR105158); *O. minuta* (IR101100, IR101128); *O. latifolia* (IR100165, IR105139) |

| | |
|---|---|
| A2414T; C2498T | |
| A1570G; C2794G | *O. officinalis* (IR101152, IR105674, IR106501); *O. australiensis* (IR105275); *O. punctata* (IR101434); *O. latifolia* (IR100165, IR105139) |
| G1607A | *O. barthii* (IR106239, IR106294, IR105990); *O. nivara* (IR92713, IR92930, IR106397, CR100400, CR100429, CR100126); *O. rufipogon* (IR80610, IR83823, IR99551, IR105491); *O. glumaepatula* (IR104387); Pusa44, Feng-Ai-Zhan, Minghui63, PR114, IR64, N22 |
| A1639G | *O. longistaminata* (IR101200, IR104301, IR105206); *O. officinalis* (IR101152, IR105674, IR106501); *O. australiensis* (IR105275); *O. punctata* (IR101434, IR105158); *O. minuta* (IR101100, IR101128); *O. latifolia* (IR100165, IR105139) |
| A1747G; A2099T; A2855G | *O. meridionalis* (IR101146); *O. longistaminata* (IR101200, IR104301, IR105206); *O. officinalis* (IR101152, IR105674, IR106501); *O. australiensis* (IR105275); *O. punctata* (IR101434, IR105158); *O. minuta* (IR101100, IR101128) |
| G1762A; A1879G | *O. officinalis* (IR101152, IR105674, IR106501); *O. australiensis* (IR105275); *O. latifolia* (IR100165, IR105139) |
| A1789T | *O. punctata* (IR101434, IR105158); *O. minuta* (IR101100, IR101128) |
| G1808A | *O. rufipogon* (IR106433) |
| C1810T | *O. meridionalis* (IR101146); *O. officinalis* (IR101152, IR105674, IR106501); *O. australiensis* (IR105275) |
| G1873A; C2087T | *O. officinalis* (IR101152, IR105674, IR106501); *O. australiensis* (IR105275) |
| G1997A | *O. nivara* (IR106397, CR100429) |
| C2077A | *O. meridionalis* (IR101146); *O. officinalis* (IR101152, IR105674, IR106501); *O. australiensis* (IR105275); *O. punctata* (IR101434, IR105158); *O. minuta* (IR101100, IR101128); *O. latifolia* (IR100165, IR105139) |
| A2084G | *O. punctata* (IR101434, IR105158) |
| A2089C | *O. officinalis* (IR101152, IR105674, IR106501); *O. australiensis* (IR105275); *O. punctata* (IR105158); *O. minuta* (IR101100, IR101128) |
| G2174A | *O. glaberrima* (IR100854 , IR101800, IR102196, IR102489, IR102512, IR102600b, IR102925, IR103750); *O. barthii* (IR100117, IR104102) |
| T2210A | *O. officinalis* (IR101152, IR106501) |
| G2232A | *O. rufipogon* (IRGC105902) |
| T2363C | *O. officinalis* (IR101152, IR105674, IR106501); *O. latifolia* (IR100165, IR105139) |
| A2456T | *O. officinalis* (IR101152, IR105674, IR106501); *O. punctata* (IR101434, IR105158); *O. minuta* (IR101100, IR101128); *O. latifolia* (IR100165, IR105139) |
| G2558T | *O. barthii* (IR106239, IR106294) |
| C2582T | *O. latifolia* (IR100165, IR105139); *O. punctata* (IR101434, IR105158) |
| G2627A | *O. latifolia* (IR100165, IR105139) |
| A2636C | *O. officinalis* (IR101152, IR105674, IR106501); *O. minuta* (IR101100, IR101128); *O. latifolia* (IR100165, IR105139) |
| A2657T; G2738A; T2795C | *O. officinalis* (IR101152, IR105674, IR106501); *O. latifolia* (IR100165, IR105139) |
| C2715T | *O. nivara* (CR100126) |
| G2727A | *O. officinalis* (IR106501) |
| **Exon3** | |
| A3407G | *O. longistaminata* (IR104301); *O. meridionalis* (IR101146); *O. officinalis* (IR101152, IR105674, IR106501); *O. australiensis* (IR105275); *O. punctata* (IR101434, IR105158); *O. minuta* (IR101100, IR101128); *O. latifolia* (IR100165, IR105139) |
| G3425A; T3446C; T3560C | *O. officinalis* (IR101152, IR105674, IR106501); *O. australiensis* (IR105275); *O. punctata* (IR101434, IR105158); *O. minuta* (IR101100, IR101128); *O. latifolia* (IR100165, IR105139) |
| C3481T | *O. meridionalis* (IR101146) |
| C3452T; T3553C; G3554A | *O. officinalis* (IR101152, IR105674, IR106501); *O. punctata* (IR101434, IR105158); *O. minuta* (IR101100, IR101128); *O. latifolia* (IR100165) |

| | |
|---|---|
| C3461T | *O. rufipogon* (IR106433, IR81976); *O. officinalis* (IR101152, IR105674, IR106501); *O. punctata* (IR101434, IR105158); *O. minuta* (IR101100, IR101128); *O. latifolia* (IR100165) |
| G3485A | *O. nivara* (IR81847) |
| C3488T | *O. officinalis* (IR101152, IR105674, IR106501) |
| A3494G; T3503G; T3563C | *O. longistaminata* (IR104301); *O. officinalis* (IR101152, IR105674, IR106501); *O. australiensis* (IR105275); *O. punctata* (IR101434, IR105158); *O. minuta* (IR101100, IR101128); *O. latifolia* (IR100165, IR105139) |
| A3497G | *O. punctata* (IR101434, IR105158); *O. minuta* (IR101100, IR101128); *O. latifolia* (IR100165, IR105139) |
| G3549A | *O. longistaminata* (IR101200, IR105206) |
| A3551C | *O. australiensis* (IR105275) |

**Table S2.** Nucleotide changes detected within the intronic region of *OsPLDα1* across the wild *Oryza* species accessions and rice cultivars in comparison to Nipponbare reference sequence.

| | |
|---|---|
| **Intron1** | |
| C172G; T183A; C196G; T226G; T227A; C229T; T230G; A234C; A235G; C236T; C238T; A240C; G245A; T246G; G247C; C248T; A253G; T255C; G256C; T264C; T265G; T268C; C269G; C272T; T274C; C275T; A280C; A281C; G282T; T283C; G286C; C289T; T290C; G291A; A292G; T294C; G305T; G307T; G309T; +T$_{302}$; -C$_{187}$; +C$_{197}$; +TT$_{249}$; +AT$_{260}$; +G$_{295}$; +GG$_{311}$; +TCGCTGTAC (222 to 230) | *O. officinalis* (IR101152, IR105674, IR106501); *O. australiensis* (IR105275); *O. minuta* (IR101100, IR101128); *O. punctata* (IR105158); *O. latifolia* (IR105139) |
| T179G; C181A; T182G; -C$_{178}$ | *O. officinalis* (IR101152, IR105674, IR106501); *O. minuta* (IR101100, IR101128); *O. punctata* (IR105158) |
| G299A; -T$_{298}$ | *O. officinalis* (IR101152, IR105674, IR106501); *O. australiensis* (IR105275); *O. punctata* (IR105158); *O. latifolia* (IR105139) |
| G308T | *O. officinalis* (IR101152, IR105674, IR106501); *O. minuta* (IR101100, IR101128) |
| T231C | *O. officinalis* (IR101152, IR105674, IR106501); |
| +ATTTCTTATCC (147 to 157) | *O. officinalis* (IR101152, IR105674, IR106501); *O. australiensis* (IR105275); *O. minuta* (IR101100, IR101128); *O. latifolia* (IR105139) |
| +ATCCTCGCTTACC (147 to 159) | *O. punctata* (IR105158) |
| -AGGTAG (176 to 181) | *O. australiensis* (IR105275); *O. latifolia* (IR105139) |
| C187T; C257G; A286T; T289C; G309T; +C$_{197}$ | *O. meridionalis* (IR101146) |
| +GCTT (229 to 232) | *O. rufipogon* (IR81976, IR104308, IR103308, IR106162) |
| +G$_{315}$ | Pusa44, Minghui63, Feng-Ai-Zhan |
| **Intron 2** | |
| T463A; C470T; C503T; -TTCT (507 to 510) | *O. longistaminata* (IR104301) |
| A467G | All selected species accessions and cultivars |
| T526C | *O. nivara* (CR100008, CR100400, CR100429, IR106397, IR81847, IR92713); *O. rufipogon* (IR89224; IR113652) |
| A575T; C800T; C897A | *O. longistaminata* (IR101200) |
| T705C | *O. nivara* (CR100400, CR100429, IR106397, IR81847); *O. rufipogon* (IR89224) |
| T749C | *O. longistaminata* (IR104301, IR101200) |
| A902G | *O. glaberrima* (IR100854 , IR101800, IR102196, IR102489, IR102512, IR102600b, IR102925, IR103750) |
| **Intron 3** | |
| C2959T | *O. nivara* (CR100008) |
| T2963C; A2978T; A2996T; A2997T; T3091C; A3115G; T3129C; G3144A; C3180T; G3201A; A3252G; A3303G; +T$_{3006}$ | *O. meridionalis* (IR101146) |
| A3006G | *O. barthii* (IR101317) |
| G3047A; C3272G | *O. glaberrima* (IR100854, IR101800, IR102196, IR102489, IR102512, IR102600b, IR102925, IR103750) |
| T3092G | *O. glaberrima* (IR100854 , IR101800, IR102196, IR102489, IR102512, IR102600b, IR102925, |

| | |
|---|---|
| | IR103750); *O. barthii* (IR100117, IR101317; IR106239) |
| A3093G | *O. barthii* (IR105990); *O. glumaepatula* (IR100184, IR104387) |
| G2903A; A2955C; G2987C; T3033C; C3045T; G3086A; C3134A; T3144C; T3147C; G3167A; A3190T; G3236A; T3338A; T3345A; A3347C; T3365G; T3372C; $+A_{3031}$; $+TA_{3096}$ +ATGCATCAGAGATCATTT (2988 to 3006) | *O. officinalis* (IR101152, IR106501); *O. punctata* (IR105158); *O. minuta* (IR101100, IR101128) |
| $+G_{3014}$ | *O. officinalis* (IR106501) |
| A3017T; T3049A; A3176T -CTAATGATCAAGCTAGTAACTTCATCTCCT (3295-3324) | *O. officinalis* (IR101152, IR106501); *O. minuta* (IR101100, IR101128) |
| A3156G; A3176C | *O. punctata* (IR105158) |

**Table S3.** qRT-PCR primers designed from different exons of *OsPLDα1*.

| Primer ID | Forward Primer (5' to 3') | Reverse Primer (5' to 3') | Amplicon size[a] |
|---|---|---|---|
| PLDE1 | AGATGCTGCTCCATGGGACG | GATGAACTTGGGGGCGCTTC | 95 bases (360-454) |
| PLDE2.1 | ACTGTGTTCTATGCCCTCGC | TGGCAACTCATGGTCAACAAC | 116 bases (1815-1930) |
| PLDE2.2 | AGTAAGATTGAAGCCGGGGAAC | ATCTCCATTGTTCTCCTTTGCCAG | 122 bases (2567-2688) |
| PLDE3 | TCATCATCGGTTCTGCAAACATC | ATCCTGTTCACCTTCTGCACAC | 223 bases (3391-3613) |

[a] Numbers in parenthesis denotes the position of bases covered by the primer pairs on *OsPLDα1* locus.

**Table S4.** Primers designed for full length amplification of *OsPLDalpha1* gene transcripts (*01t0172400-1*, *01t0172400-4*, and *01t0172400-5*).

| Primer ID | Forward Primer (5' to 3') | Reverse Primer (5' to 3') | Amplicon size |
|---|---|---|---|
| PLDT01 | CAAGTTTGTGGAGGGGATTGAGG | AACACATCATCCAGCATTCCCAG | 2094 bases (105-2198) |
| PLDT04 | GTTCTATGCCCTCGCAACCC | ACACATCATCCAGCATTCCCA | 1280 bases (34-1303) |
| PLDT05 | GGGTGTTCCAGAGAGTGGAT | CAGCTCAGTCACCACACCATC | 622 bases (63-684) |
